# A Novel Agonist of the TRIF Pathway Induces a Cellular State Refractory to Replication of Zika, Chikungunya, and Dengue Viruses

**DOI:** 10.1128/mBio.00452-17

**Published:** 2017-05-02

**Authors:** Kara M. Pryke, Jinu Abraham, Tina M. Sali, Bryan J. Gall, Iris Archer, Andrew Liu, Shelly Bambina, Jason Baird, Michael Gough, Marita Chakhtoura, Elias K. Haddad, Ilsa T. Kirby, Aaron Nilsen, Daniel N. Streblow, Alec J. Hirsch, Jessica L. Smith, Victor R. DeFilippis

**Affiliations:** a Vaccine and Gene Therapy Institute, Oregon Health and Science University, Portland, Oregon, USA; b Veterans Affairs Medical Center, Portland, Oregon, USA; c Earle A. Chiles Research Institute, Robert W. Franz Cancer Center, Providence Portland Medical Center, Portland, Oregon, USA; d Division of Infectious Diseases and HIV Medicine, Drexel College of Medicine, Philadelphia, Pennsylvania, USA; University of Pittsburgh School of Medicine

**Keywords:** antiviral agents, emerging virus, innate immunity, interferons

## Abstract

The ongoing concurrent outbreaks of Zika, Chikungunya, and dengue viruses in Latin America and the Caribbean highlight the need for development of broad-spectrum antiviral treatments. The type I interferon (IFN) system has evolved in vertebrates to generate tissue responses that actively block replication of multiple known and potentially zoonotic viruses. As such, its control and activation through pharmacological agents may represent a novel therapeutic strategy for simultaneously impairing growth of multiple virus types and rendering host populations resistant to virus spread. In light of this strategy’s potential, we undertook a screen to identify novel interferon-activating small molecules. Here, we describe 1-(2-fluorophenyl)-2-(5-isopropyl-1,3,4-thiadiazol-2-yl)-1,2-dihydrochromeno[2,3-*c*]pyrrole-3,9-dione, which we termed AV-C. Treatment of human cells with AV-C activates innate and interferon-associated responses that strongly inhibit replication of Zika, Chikungunya, and dengue viruses. By utilizing genome editing, we investigated the host proteins essential to AV-C-induced cellular states. This showed that the compound requires a TRIF-dependent signaling cascade that culminates in IFN regulatory factor 3 (IRF3)-dependent expression and secretion of type I interferon to elicit antiviral responses. The other canonical IRF3-terminal adaptor proteins STING and IPS-1/MAVS were dispensable for AV-C-induced phenotypes. However, our work revealed an important inhibitory role for IPS-1/MAVS, but not TRIF, in flavivirus replication, implying that TRIF-directed viral evasion may not occur. Additionally, we show that in response to AV-C, primary human peripheral blood mononuclear cells secrete proinflammatory cytokines that are linked with establishment of adaptive immunity to viral pathogens. Ultimately, synthetic innate immune activators such as AV-C may serve multiple therapeutic purposes, including direct antimicrobial responses and facilitation of pathogen-directed adaptive immunity.

## IMPORTANCE

The type I interferon system is part of the innate immune response that has evolved in vertebrates as a first line of broad-spectrum immunological defense against an unknowable diversity of microbial, especially viral, pathogens. Here, we characterize a novel small molecule that artificially activates this response and in so doing generates a cellular state antagonistic to growth of currently emerging viruses: Zika virus, Chikungunya virus, and dengue virus. We also show that this molecule is capable of eliciting cellular responses that are predictive of establishment of adaptive immunity. As such, this agent may represent a powerful and multipronged therapeutic tool to combat emerging and other viral diseases.

## INTRODUCTION

The spontaneity and clinical impact of emerging mosquito-transmitted viral pathogens are illustrated exceptionally well by the recent appearance of Chikungunya virus (CHIKV) and Zika virus (ZIKV) in Latin America and the Caribbean (1). The preexisting transmission of dengue virus (DENV) across this geographic region (2) has resulted in cocirculation of three substantially pathogenic arboviruses that exhibit similar acute symptomatology for which long-term sequelae are also known (3, 4). This cocirculation not only renders differential diagnoses more complicated (5) but also may lead to enhanced but unknown disease manifestations during viral coinfection (6). Unfortunately, no FDA-approved antiviral treatments are currently available for any of these agents, and vaccines are only in use for DENV.

CHIKV is an *Alphavirus* that first emerged in the Americas in late 2013 (7) and has infected over a million people in the region since then (8). Acute infection is associated with febrile illness and debilitating joint pains but may also induce central nervous system disease, especially in neonates. While immunity is believed to be lifelong, severe joint pain may persist for months to years (9). ZIKV is a member of the *Flaviviridae* and dramatically emerged in the Americas in 2015 and 2016. As of this writing, 50 countries in the Americas have reported autochthonous transmission of the virus (http://www.cdc.gov/zika/geo/active-countries.html). The World Health Organization (WHO) has estimated that 3 to 4 million individuals will be infected with ZIKV in the coming year (10). It is estimated that 80% of acute ZIKV infections are asymptomatic, with the remaining 20% clinically resembling infection by CHIKV and DENV, including fever, rash, headache, and arthralgia, although ZIKV appears to be distinctly associated with conjunctivitis (11). Neurological complications, including Guillain-Barré syndrome, have also been reported following ZIKV infection (12). Most importantly, however, ZIKV infection during pregnancy is associated with severe teratogenic effects, including microcephaly (13). As such, the need for prophylactic and therapeutic strategies to combat these pathogens is extremely high.

The type I interferon (IFN) system represents an antiviral response that is shared by all vertebrates and has thus been evolutionarily shaped by exposure through millennia to an unknowable diversity of pathogens (14). The outcome is a cell-based protective response that exhibits efficacy against a phylogenetically broad range of viruses that are both ubiquitous and potentially harmful. Type I IFNs (multiple IFN-α subtypes and IFN-β) are cytokines that bind the IFN-α receptor (IFNAR) present on nearly all cells and induce signaling via Janus kinases (JAKs) to the protein signal transducer and activator of transcription 1 (STAT1) and STAT2. These proteins activate synthesis of hundreds of IFN-stimulated genes (ISGs), many of which confer direct antiviral effects by generating a cellular state antagonistic to virus growth, while others facilitate and coordinate adaptive immune responses (15). IFNs themselves are transcribed and secreted in response to cellular detection of pathogen-associated molecular patterns (PAMPs). This process is triggered by pattern recognition receptors (PRRs) that, in response to PAMP engagement, initiate signaling cascades that culminate in the activation of transcription factors such as IFN regulatory factor 3 (IRF3) and nuclear factor κB (NF-κB) (reviewed in reference 16). These, in turn, transcribe IFNs as well as other proinflammatory cytokines and antiviral effectors. Three principal PRR-driven, IRF3-terminal pathways defined by their unique adaptor proteins are known and include IFN promoter stimulator 1 (IPS-1, also known as MAVS, VISA, or CARDIF), which integrates signaling from the PRRs of cytoplasmic double-stranded RNA (dsRNA) RIG-I and MDA5 (17–20), the stimulator of IFN genes protein (STING; also known as MITA, ERIS, MPYS, or TMEM173), which is both a PRR for cyclic dinucleotides and an adaptor for PRRs of cytoplasmic dsDNA (21–25), and Toll/interleukin-1 (IL-1) receptor domain-containing adapter activating IFN (TRIF, also known as TICAM), which transmits signaling from Toll-like receptor 3 (TLR3) and TLR4 (26, 27). Together, these pathways are capable of stimulating the IFN response following exposure to a wide array of molecular indicators of microbial infection.

Given the phenotypic potency and target breadth of the type I IFN system, it has been utilized as a host-directed method to block virus infection. In fact, treatment with various forms of IFN itself has demonstrated clinical success against hepatitis B and C viruses (28–30). However, use of systemic IFNs is associated with substantial undesirable effects. For instance, extraordinarily high doses of IFN, such as those administered during conventional therapy, are associated with multiple potentially severe side effects, most notably neurotoxicity and neuropsychiatric complications (31, 32) that contribute to voluntary cessation of treatment. Additionally, clinical-grade IFN is costly to produce and requires repeated delivery due to a short *in vivo* half-life, and these factors contribute to a high overall expense. Alternatively, pharmacological induction of IFN-dependent responses has been pursued as a strategy to combat viral infection (33). Experimental induction of IFN synthesis via administration of PAMPs or small molecules has also proven efficacious against multiple virus types *in vitro* and *in vivo* (34–40). This includes recent work from our group in which we described a novel small-molecule agonist of the human STING pathway that elicits antiviral effects against alphaviruses, including CHIKV (41). Furthermore, targeted activation of innate cytokine responses can also be harnessed for other therapeutic outcomes, such as vaccine adjuvanticity (42) and antitumor responses (43), thus providing multiple potential clinical uses. Importantly, given that the effects of IFN are broad with respect to known viral pathogens, it is virtually certain that numerous unknown, potentially emerging, and zoonotic virus types are similarly susceptible to IFN-induced cellular states. As such, pharmacological IFN activation may represent a cost-effective and impactful antiviral strategy applicable for populations prone to virus emergence events. Moreover, the ability to simultaneously impair growth of multiple virus types by using a single therapy would be attractive in areas where transmission of multiple viruses is ongoing, such as current-day Latin America and the Caribbean. In light of this, we describe here a novel small molecule capable of rendering human cells inhibitory to growth of ZIKV, CHIKV, and DENV and of eliciting proinflammatory responses from human immune cells. By using deeper molecular analysis, we show that the cellular target of this compound is the IRF3-terminal TRIF pathway.

## RESULTS

### Compound AV-C induces type I IFN-dependent transcription in human fibroblasts.

**FIG 1:**
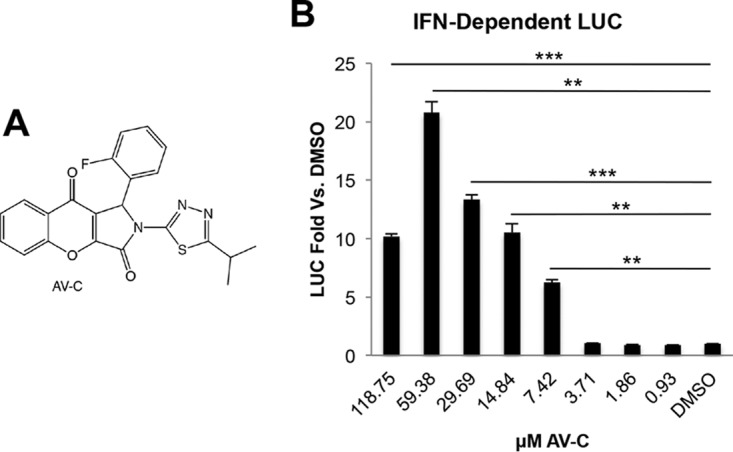
Activation of type I interferon-associated transcription after exposure to AV-C. (A) Chemical structure of 1-(2-fluorophenyl)-2-(5-isopropyl-1,3,4-thiadiazol-2-yl)-1,2-dihydrochromeno[2,3-*c*]pyrrole-3,9-dione (AV-C). (B) Reporter assay results, showing induction of ISRE-dependent LUC expression in THF-ISRE cells at 8 h posttreatment at the indicated concentrations. Values displayed are average fold changes ± standard deviations, based on three replicates in comparison to results in DMSO-treated cells. Unpaired-sample Student’s *t* test comparisons were made between AV-C- and DMSO-treated cells. **, *P* ≤ 0.01; ***, *P* ≤ 0.001.

To identify novel small molecules capable of activating innate immune responses in human cells, we employed foreskin fibroblasts stably transfected with constitutively expressed human telomerase reverse transcriptase for prevention of senescence (44). The luciferase (LUC) open reading frame from *Phytonis pyralis* downstream of a promoter element that is activated by type I IFN-mediated cell signaling was stably introduced by lentiviral transduction (THF-ISRE), as described elsewhere (45). Using these cells in a high-throughput screening platform, we examined approximately 51,000 chemically diverse compounds in duplicate for their ability to significantly stimulate LUC expression (41). One stimulatory molecule was 1-(2-fluorophenyl)-2-(5-isopropyl-1,3,4-thiadiazol-2-l)-1,2-dihydrochromeno[2,3-*c*]pyrrole-3,9-dione, which we termed AV-C (Fig. 1A; see also Fig. S1 in the supplemental material). We next performed a follow-up experiment to both validate the screening results and examine whether dose-dependent LUC synthesis occurred. As shown in Fig. 1B, exposure of THF-ISRE cells to media containing AV-C resulted in concentration-associated expression of IFN-dependent LUC. These results suggest that the molecule triggers an innate cellular response that stimulates IFN-dependent signaling in human fibroblasts.

10.1128/mBio.00452-17.1 FIG S1  High-throughput identification of AV-C. Luciferase (LUC) expression of the top activating molecules as described in the text from a high-throughput screen of 51,632 compounds. Presented values represent the average ± standard deviation of results from duplicate assays. The black bar and arrow indicate the LUC signal generated by AV-C. Download FIG S1, PDF file, 0.1 MB. Copyright © 2017 Pryke et al. 2017 Pryke et al. This content is distributed under the terms of the Creative Commons Attribution 4.0 International license.

### AV-C activates transcription and translation of antiviral effector genes.

**FIG 2:**
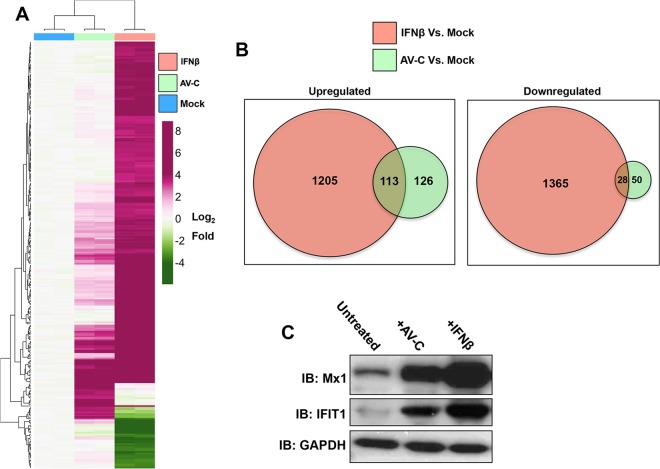
AV-C-mediated induction of IFN-stimulated antiviral mRNA and proteins. (A) Heat map illustrating signals from individual hybridization array probes relative to the mean composite signal for all treatments of THF cells (untreated mock, 1,000 U/ml IFN-β, or 25 μM AV-C). Data presented are expressed as the log_2_ fold difference, and only probes found to be significantly increased or decreased following any treatment are presented. (B) Venn diagram illustrating numbers of annotated RNAs significantly up- or downregulated relative to results with mock-treated cells for AV-C- or IFN-β-treated cells. (C) Immunoblot results for IFN-stimulated antiviral proteins IFIT1 and Mx1 in THFs left untreated or treated with AV-C (25 μM) or IFN-β (1,000 U/ml) for 8 h.

Since AV-C efficiently activated expression of a heterologous IFN-dependent reporter, we next examined the degree to which the molecule could also induce transcription of endogenous genes, in particular those that are known to be downstream of IFN pathways. For this, we utilized genomic hybridization-based microarrays. THF cells were treated in duplicate with 1% dimethyl sulfoxide (DMSO; mock treatment), 25 μM AV-C, or 1,000 U/ml IFN-β for 8 h. Total RNA was harvested, and probes complementary to mRNA were synthesized and hybridized to Affymetrix Primeview microarrays as described in Materials and Methods. Probe sets exhibiting signals from AV-C or IFN-β treatments that were significantly above or below those from mock-treated cells were then statistically retrieved using analysis of variance (ANOVA) as implemented within the Affymetrix transcriptome analysis console. Figure 2A displays in heat map format all individual probe sets from each treatment that were found to exhibit differential signals relative to mock-treated cells following exposure to either AV-C or IFN-β. The Venn diagrams in Fig. 2B illustrate that 113 annotated coding regions were co-upregulated and 28 were co-downregulated in response to AV-C and IFN-β treatments. Moreover, 1,205 and 126 genes were uniquely upregulated in response to IFN-β and AV-C treatments, respectively, and 1,365 and 50 genes were uniquely downregulated in response to IFN-β and AV-C treatments, respectively. Individual fold changes for all regulated genes are included in Table S1. Interestingly, among the mRNAs coinduced between AV-C and IFN-β treatments were many known to encode proteins that confer directly antiviral phenotypes, including Mx1 (46), IFIT1 (47), RSAD2/Viperin (48), and OAS1 (49). Moreover, microarray results indicated that AV-C induced the transcription of IFN-β itself which, if translated and secreted, would presumably lead to ISG expression via autocrine/paracrine signaling. Intriguingly, other immune-associated mRNAs that are involved in inflammatory and adaptive immune responses were exclusively induced by treatment with AV-C but not IFN-β, including PTGS2/COX-2 (50), CCL5 (51), CXCL8/IL-8 (52), and IL-6 (53). We next examined whether translation of coregulated antiviral proteins was also detectable in response to treatment with AV-C, a requirement for establishment of an antiviral cellular state. As shown in Fig. 2C, both IFN-β and AV-C triggered the synthesis of antiviral proteins Mx1 and IFIT1, suggesting that AV-C is likely to alter the innate phenotypic condition of cells exposed to it. Whether the transcriptional induction program generated by exposure to AV-C is sufficient to block virus replication was next investigated in light of these findings.

10.1128/mBio.00452-17.9 TABLE S1  Absolute fold changes relative to overall mean signals in mock-treated cells of individual Affymetrix probe sets found to be differentially regulated following treatment with either AV-C or IFN-β. Download TABLE S1, XLS file, 0.3 MB. Copyright © 2017 Pryke et al. 2017 Pryke et al. This content is distributed under the terms of the Creative Commons Attribution 4.0 International license.

### AV-C elicits a cellular state that inhibits replication of ZIKV, CHIKV, and DENV.

**FIG 3:**
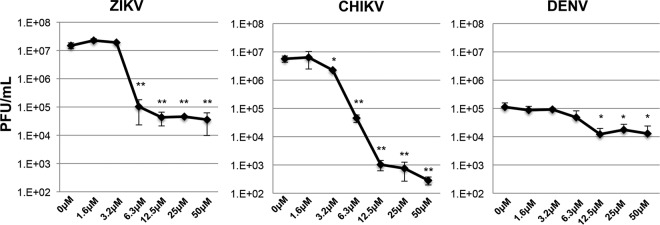
AV-C elicits a cellular state refractory to virus replication. Average results (PFU per milliliter, ± the standard deviation) of ZIKV, CHIKV, and DENV grown on THF cells in triplicate in the presence of the indicated AV-C concentrations (the DMSO concentration was normalized to 1%) added 6 h preinfection. Unpaired-sample Student’s *t* test comparisons were made between AV-C- and DMSO-treated cells. **, *P* ≤ 0.01; ***, *P* ≤ 0.001.

Based on our observations that AV-C activates transcription and translation of known antiviral effector genes as well as IFN-β itself, we hypothesized that cells preexposed to the compound would be refractory to virus growth. To address this, we examined replication of CHIKV, ZIKV, and DENV on AV-C-treated fibroblasts; these three geographically cooccurring mosquito-transmitted viruses are of recent and extraordinary clinical importance (1, 54). Human fibroblasts represent a permissive and crucial cell type for CHIKV and ZIKV and thus constitute a biologically relevant *in vitro* model for these pathogens (55, 56). THF cells were first pretreated with AV-C over a range of molarities for 6 h. Cells were then infected with CHIKV, ZIKV, or DENV in the presence of AV-C for the indicated durations. As shown in Fig. 3, treatment with AV-C diminished replication of CHIKV and ZIKV by multiple logs, displaying 90% inhibitory concentrations (IC_90_) of 5.815 μM (ZIKV) and 3.538 μM (CHIKV), which are well below dosages observed to induce detectable cytotoxicity (Fig. S2). DENV, in contrast, did not grow to comparable peak titers on these cells, an observation we hypothesize is due to the virus’ relative inability to counteract existing antiviral innate signaling in these cells (see below). Nevertheless, DENV still showed sensitivity to AV-C, with an IC_90_ of 9.939 μM. Based on these results, we concluded that, despite not inducing a fully overlapping subset of IFN-stimulated genes (Fig. 2), AV-C treatment nevertheless generates a cellular state that is antagonistic to replication of DENV, CHIKV, and ZIKV. Among these viruses, CHIKV clearly demonstrated the highest degree of susceptibility to the AV-C-induced cellular state, with an approximately 4-log peak reduction in virus titer. ZIKV was also highly sensitive, however, exhibiting an approximately 2.5-log titer reduction.

10.1128/mBio.00452-17.2 FIG S2  ATP-dependent luminescence of THF following 24-h or 48-h exposure to the indicated concentrations of AV-C (with the DMSO concentration normalized to 3%). Values displayed are raw luminescence values averaged from quadruplicate measurements (± standard deviations) following 24-h or 48-h exposure to the indicated concentration of AV-C. Download FIG S2, PDF file, 0.1 MB. Copyright © 2017 Pryke et al. 2017 Pryke et al. This content is distributed under the terms of the Creative Commons Attribution 4.0 International license.

Alphaviruses and flaviviruses possess evolutionarily analogous yet biologically potent phenotypes to counteract the antiviral effects of IFN stimulation (reviewed in references 57 and 58). We therefore asked whether AV-C could inhibit virus growth when it was added to cells after infection, a circumstance that would potentially allow its use in therapeutic treatment. To address this, we exposed THF-ISRE cells to AV-C at different times relative to viral inoculation. As shown in Fig. S3, AV-C lost its ability to block CHIKV growth when added 2 h postinfection, a finding consistent with the appearance of alphavirus innate evasion proteins (59). Since ZIKV exhibited higher overall sensitivity to AV-C than did DENV (Fig. 3), likely an effect of cell permissivity, we examined the effect of AV-C added postinfection on the growth of a representative flavivirus. In this case, inhibition by AV-C was evident as late as 16 h postinfection, indicating either that the compound exhibits antiviral effects that are virus type specific or that the ZIKV IFN evasion phenotypes are expressed much later in infection than those against CHIKV.

10.1128/mBio.00452-17.3 FIG S3  Effect of AV-C time of addition on virus replication. Average results (in PFU per milliliter ± the standard deviation) are shown for ZIKV and CHIKV grown on THF cells in triplicate in the presence of 12.5 μM AV-C (the DMSO concentration was normalized to 1%) added to cells at the indicated times pre- or postinfection. Medium was harvested at 48 h (CHIKV) or 72 h (DENV and ZIKV) postinfection, and titers were determined based on serial dilution PFU (CHIKV) or FFU (ZIKV and DENV) assay on Vero cells. Download FIG S3, PDF file, 0.1 MB. Copyright © 2017 Pryke et al. 2017 Pryke et al. This content is distributed under the terms of the Creative Commons Attribution 4.0 International license.

### AV-C-induced gene expression requires IRF3 phosphorylation and canonical IRF3 kinase activity.

**FIG 4:**
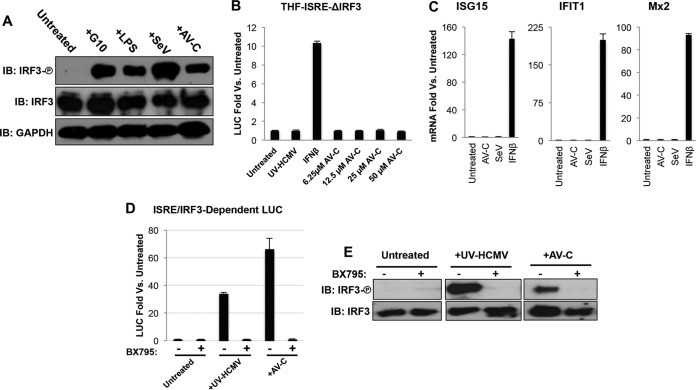
AV-C induces canonical IRF3 activation and IRF3- and IFN-dependent transcription. (A) Immunoblot results, showing S386 phosphorylation status of IRF3, total IRF3, and GAPDH from THF lysates following 4-h treatment with 1% DMSO (untreated), 100 μM G10, 1 ng/ml LPS, 160 HAU/ml SeV, or 25 μM AV-C. (B) Reporter assay results, showing induction of ISRE-dependent LUC expression in THF-ISRE cells lacking IRF3 following 8-h treatment with UV-inactivated HCMV (MOI, 3), 1,000 U/ml IFN-β, or AV-C at the indicated concentrations. Values displayed are average fold changes versus results in DMSO-treated cells of three replicates, ± the standard deviation (SD). (C) Transcription of ISG15, IFIT1, and Mx2 in cells treated for 8 h with 25 μM AV-C, SeV (160 HAU/ml), or 1,000 U/ml IFN-β. Values displayed are average fold changes versus results in DMSO-treated cells of two biological replicates, ± SD. (D) Reporter assay showing induction of LUC expression in THF-ISRE cells left untreated or exposed to UV-HCMV (MOI, 3) or 25 μM AV-C in the presence or absence of the TBK1/IKKi inhibitor BX795 (10 nM). Values displayed are average fold changes versus results with DMSO-treated cells of three replicates, ± SD. (E) Immunoblot results, showing the S386 phosphorylation status of IRF3 as well as total IRF3 in THFs left untreated following 4-h exposure to UV-HCMV (MOI, 3) or 25 μM AV-C in the presence or absence of 10 nM BX795.

AV-C stimulates expression of genes that are downstream of promoters activated by type I IFN as well as the IFN-β gene itself, and IRF3 is conventionally required for IFN synthesis. To begin to dissect the molecular basis of host cell responses to and targets of AV-C, we next investigated whether IRF3 is involved in innate activation by AV-C. First, we examined the phosphorylation status of IRF3 in response to AV-C exposure. As shown in Fig. 4A, treatment of THF cells with stimuli activating the three key IRF3-terminal signaling pathways (discussed in more detail below) defined by the adaptor proteins IPS-1/MAVS (Sendai virus [SeV] [20]), STING (molecule G10 [41]), and TRIF (lipopolysaccharide [LPS] [60]), all activated IRF3 phosphorylation at 4 h posttreatment. Similarly, AV-C at 25 μM also led to IRF3 phosphorylation in these cells, as well as numerous additional human cell types, including HeLa cells, HEK-293 cells, SK-N-MC neuronal cells, THP-1 promonocytic cells, and microvascular endothelial cells (Fig. S4). We next investigated whether IRF3 is functionally essential to AV-C-induced gene transcription. For this, we employed previously described THF-ISRE cells from which IRF3 was deleted via CRISPR/Cas9 technology (THF-ISRE-ΔIRF3) (41). As shown in Fig. 4B, no detectable LUC expression was observed in these cells following treatment with multiple concentrations of AV-C or human cytomegalovirus particles rendered inactive by UV treatment (UV-HCMV), a STING-inducing stimulus (61). Importantly, IFN-dependent JAK/STAT signaling was operational in these cells, as demonstrated by LUC expression in response to IFN-β exposure. We next verified that AV-C-mediated induction of endogenous genes was similarly sensitive to the presence of IRF3. For this, semiquantitative reverse transcriptase (RT)-PCR (qPCR) was used to measure mRNA synthesis in THF-ISRE-ΔIRF3 cells following treatment with AV-C, SeV, or IFN-β relative to synthesis in cells treated with 1% DMSO. Figure 4C shows that only IFN-β stimulation led to mRNA induction of ISG15, IFIT1, and Mx2 and that no induction was seen with AV-C or the IPS-1/MAVS-activating stimulus SeV. IRF3-dependent transcription requires phosphorylation-mediated activation of the protein by TANK binding kinase 1 (TBK1) or inducible I-kappa B kinase epsilon (IKKε) (62). We therefore next examined whether AV-C activates canonical TBK1- or IKKε-dependent IRF3 phosphorylation. THF-ISRE cells were treated with AV-C or UV-HCMV in the presence and absence of BX795, a small-molecule inhibitor of TBK1 and IKKε kinase activities (63). As shown in Fig. 4D, treatment of THF-ISRE cells with UV-HCMV or AV-C in the presence, but not absence, of BX795 failed to stimulate LUC expression. Moreover, the immunoblotting results in Fig. 4E illustrate that exposure of cells to UV-HCMV or AV-C activated phosphorylation of IRF3, a process that does not occur in the presence of BX795. These results indicate that AV-C acts upstream of TBK1/IKKε-mediated phosphorylation of IRF3 and subsequent IRF3-dependent gene transcription.

10.1128/mBio.00452-17.4 FIG S4  Immunoblots showing the phosphorylation status of IRF3 S386 and GAPDH (loading control) in HeLa, SK-N-M.C., HEK-293, THP-1, and TDMV-B cells either left untreated or following 4 h treatment with 25 μM AV-C. Download FIG S4, PDF file, 0.2 MB. Copyright © 2017 Pryke et al. 2017 Pryke et al. This content is distributed under the terms of the Creative Commons Attribution 4.0 International license.

### AV-C-mediated, IRF3-dependent innate activation occurs independently of adaptor proteins IPS-1/MAVS and STING but requires TRIF.

Conventional IRF3-terminal signaling pathways lead from PRRs via adaptor proteins to TBK1/IKKε kinase and IRF3 activation. The adaptors include IPS-1/MAVS, which is required for signals initiated by the cytoplasmic dsRNA receptors RIG-I and MDA5 (17–20), STING, which is both a cytoplasmic PRR of cyclic dinucleotides and also an adaptor for cytoplasmic receptors of dsDNA (23–25, 64), and TRIF, which is essential for signaling initiated from TLR3 and TLR4, which sense dsRNA and LPS, respectively. Since all three are widely known to be essential components of defined signaling cascades that culminate in IRF3 phosphorylation, we hypothesized that at least one is required for innate stimulation by AV-C. To address this, we used previously described THF-ISRE cells lacking IPS-1/MAVS or STING (termed THF-ISRE-ΔIPS1 and THF-ISRE-ΔSTING cells) (41) as well as newly constructed cells lacking TRIF (THF-ISRE-ΔTRIF cells).

**FIG 5:**
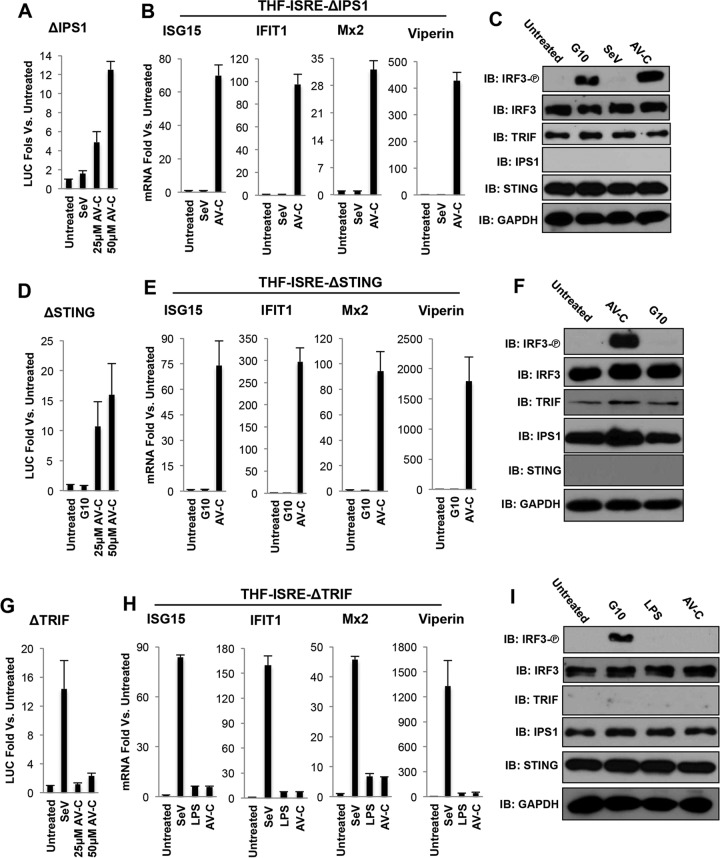
AV-C induces IRF3-mediated transcriptional activity in a manner independent of STING and IPS-1/MAVS but dependent on TRIF. (A) Reporter assay results, showing induction of ISRE-dependent LUC expression in THF-ISRE cells lacking IPS1/MAVS (THF-ISRE-ΔIPS1) following 8-h treatment with SeV (160 HAU/ml) or AV-C at the indicated concentrations. Values displayed are average fold changes compared to results with DMSO-treated cells of three replicates (± standard deviations [SD]). (B) Transcription of ISG15, IFIT1, viperin, and Mx2 in THF-ISRE-ΔIPS1 cells treated for 8 h with SeV (160 HAU/ml), or 50 μM AV-C. Values displayed are average fold changes versus results in DMSO-treated cells of two biological replicates, ± SD. (C) Immunoblot showing S386 phosphorylation status of IRF3 as well as total IRF3, TRIF, STING, IPS1/MAVS, and GAPDH in THF-ISRE-ΔIPS1 cells left untreated and following 4-h exposure to 100 μM G10, SeV (160 HAU/ml), or 25 μM AV-C. (D) Reporter assay results, showing induction of ISRE-dependent LUC expression in THF-ISRE cells lacking STING (THF-ISRE-ΔSTING) following 8-h treatment with 100 μM G10 or AV-C at the indicated concentrations. Values displayed are average fold changes compared to results in DMSO-treated cells of three replicates, ± SD. (E) Transcription of ISG15, IFIT1, viperin, and Mx2 in THF-ISRE-ΔSTING cells treated for 8 h with 100 μM G10 or 25 μM AV-C. Values displayed are average fold changes compared to results in DMSO-treated cells of two biological replicates, ± SD. (F) Immunoblot results showing the S386 phosphorylation status of IRF3 as well as total IRF3, TRIF, STING, IPS1/MAVS, and GAPDH in THF-ISRE-ΔSTING cells left untreated and following 4-h exposure to 100 μM G10 or 25 μM AV-C. (G) Reporter assay results, showing induction of ISRE-dependent LUC expression in THF-ISRE cells lacking TRIF (THF-ISRE-ΔTRIF) following 8-h treatment with 100 μM G10 or AV-C at the indicated concentrations. Values displayed are average fold changes compared to results in DMSO-treated cells of three replicates, ± SD. (H) Transcription of ISG15, IFIT1, viperin, and Mx2 in THF-ISRE-ΔTRIF cells treated for 8 h with 100 μM G10 or 25 μM AV-C. Values displayed are average fold changes versus results in DMSO-treated cells of two biological replicates, ± SD. (I) Immunoblot results, showing S386 phosphorylation status of IRF3 as well as total IRF3, TRIF, STING, IPS1/MAVS, and GAPDH in THF-ISRE-ΔTRIF cells left untreated and following 4-h exposure to 100 μM G10 or 25 μM AV-C.

As shown in Fig. 5, AV-C, but not the control stimulus SeV, was able to induce IFN-dependent LUC expression in THF-ISRE-ΔIPS1 cells. Furthermore, AV-C treatment also led to transcription of endogenous antiviral mRNAs for ISG15, IFIT1, Mx2, and viperin, whereas SeV treatment, as expected, did not result in transcription in the absence of IPS1/MAVS (Fig. 5B). Consistent with these results, AV-C was also able to activate IRF3 phosphorylation in cells lacking IPS-1/MAVS (Fig. 5C), as was the STING pathway agonist G10 (41), but not SeV. AV-C also induced expression of IFN-dependent LUC in cells lacking STING, in contrast to results with the control compound G10 (Fig. 5D). Additionally, AV-C, but not G10, was able to stimulate transcription of ISG15, IFIT1, Mx2, and viperin mRNAs (Fig. 5E), as well as IRF3 phosphorylation (Fig. 5F) in THF-ISRE-ΔSTING cells.

We next examined whether AV-C triggered innate induction in the absence of TRIF. As shown in Fig. 5G, while the IPS-1/MAVS-activating stimulus SeV was able to trigger substantial LUC signal in THF-ISRE-ΔTRIF cells, treatment with AV-C at 25 μM or 50 μM did not. Similarly, strong synthesis of ISG15, IFIT1, viperin, and Mx2 mRNA was observed in these cells following treatment with SeV but not with AV-C or the TLR4/TRIF agonist LPS (Fig. 5H). Consistent with these results, phosphorylation of IRF3 was only detected in THF-ISRE-ΔTRIF cells following treatment with SeV but not AV-C or LPS (Fig. 5I). Based on these observations, we conclude that the IRF3-terminal innate response induced by AV-C occurs via a STING- and IPS1/MAVS-independent but TRIF-dependent signaling pathway.

### AV-C-induced antiviral activity requires TRIF- and IFNAR-dependent signaling.

**FIG 6:**
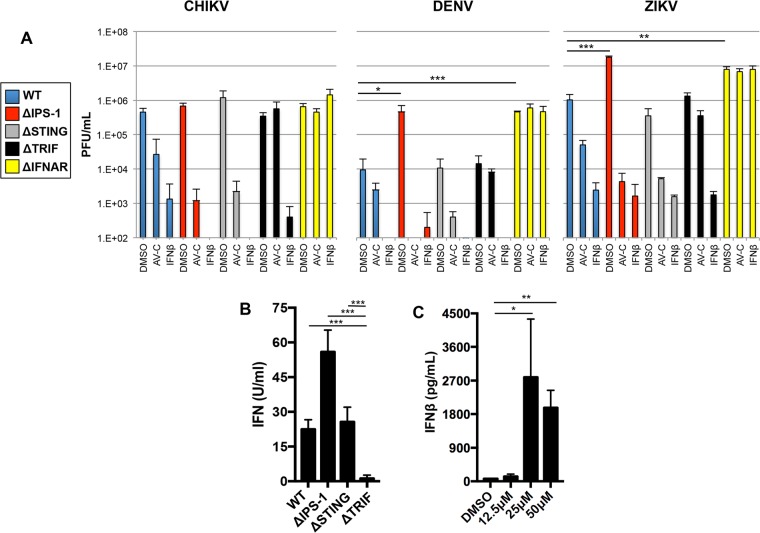
AV-C elicits an antiviral state in cells lacking the IPS-1/MAVS or STING pathways but not the TRIF or IFNAR pathways. (A) Average PFU per milliliter (± the standard deviation) for CHIKV, DENV, or ZIKV grown on wild-type (WT) THF-ISRE cells (blue) and THF-ISRE lacking IPS-1/MAVS (red), STING (gray), TRIF (black), or IFNAR (yellow) following 6 h pretreatment with 1% DMSO, 12.5 μM AV-C, or 1,000 U/ml IFN-β (the DMSO concentration was normalized to 1%). Infections were performed in triplicate, and virus titers were determined by serial dilution plaque assay at 48 h postinfection (CHIKV) or by FFU assay at 72 h postinfection (ZIKV and DENV) as described in the text. (B) Average units per milliliter of type I IFN, as determined in a luciferase bioassay of levels secreted from the indicated cells following 8-h treatment with 12.5 μM AV-C. (C) Average (in picograms per milliliter) ± the standard deviation of secreted IFN-β, determined by ELISA from human PBMCs following 8-h treatment in triplicate with the indicated concentrations of AV-C. An unpaired Student’s *t* test was used to determine statistical significance. *, *P* < 0.05; **, *P* < 0.01; ***, *P* < 0.001.

Our results indicated that IRF3-mediated responses elicited by AV-C, including expression of antiviral effectors, occurs in a manner dependent on TRIF but not IPS-1/MAVS or STING. We therefore examined whether similar functional relationships are evident with respect to antiviral cellular states induced by the molecule. We therefore pretreated THF-ISRE, THF-ISRE-ΔIPS1, THF-ISRE-ΔSTING, and THF-ISRE-ΔTRIF cells with AV-C at 12.5 μM, which is over twice the IC_90_ for ZIKV, over 3.5 times the IC_90_ for CHIKV, and 1.25 times the IC_90_ for DENV on wild-type cells (Fig. 3). We also used IFN-β as a positive control and DMSO as the negative control and infected treated cells with CHIKV, DENV, or ZIKV, as described above. As shown in Fig. 6A, replication of the three virus types was strongly impaired in all four mutant cell lines pretreated with IFN-β, indicating that their JAK/STAT-dependent antiviral responses were appropriately intact. In addition, AV-C pretreatment of THF-ISRE, THF-ISRE-ΔIPS1, and THF-ISRE-ΔSTING cells also significantly diminished virus replication relative to that with the negative-control treatment, in agreement with the innate activation states presented in Fig. 5. Viral growth in AV-C-treated cells lacking TRIF, however, appeared statistically similar to that observed following mock treatment of these cells. This observation also agrees with the identified role for TRIF in IRF3-mediated activity and shows that this relationship extends to the molecule’s elicitation of an antiviral state.

Given the profound susceptibility of CHIKV, DENV, and ZIKV to IFN-induced cellular states and the functional link between IRF3 activation and IFN synthesis, we decided to additionally ask whether AV-C-induced, TRIF-dependent antiviral activity also required IFN-dependent signaling. Moreover, while AV-C induces transcription of IFN-β (Fig. 2), it also stimulates expression of ISGs such as ISG15, IFIT1, and viperin, which confer antiviral activity but can also be induced by IRF3 alone, in the absence of IFN (65, 66). To address this, we used CRISPR/Cas9 to construct THF-ISRE cells lacking the type I IFN receptor IFNAR (THF-ISRE-ΔIFNAR cells). As shown in Fig. 6A, pretreatment of these cells with IFN-β had no effect on the growth of any of the three viruses relative to control pretreatment, phenotypically validating the IFNAR deletion. Interestingly, AV-C pretreatment also failed to elicit a block to virus replication in these cells (Fig. 6A), indicating that the AV-C-mediated antiviral state may be primarily conferred by autocrine/paracrine signaling mediated by TRIF-dependent synthesis of type I IFN. We therefore examined the ability of AV-C to trigger secretion of type I IFN from exposed cells. For this, we first utilized a cellular LUC-based bioassay to measure subtype-independent bioactive IFN in media harvested from AV-C-treated cells. Figure 6B shows that the compound was able to induce IFN secretion from wild-type fibroblast cells as well as those lacking IPS-1/MAVS or STING but not TRIF, in agreement with our results described above. In addition, AV-C was also capable of inducing secretion of IFN-β from human peripheral blood mononuclear cells (PBMCs) (Fig. 6C), indicating that this effect is likely cell type independent. Based on these results, we conclude that the antiviral cellular state elicited by AV-C requires TRIF-dependent secretion of type I IFN and subsequent IFNAR-mediated expression of antiviral effectors.

An unexpected finding from these experiments was that the replication of DENV in cells lacking IPS-1/MAVS or IFNAR was >1.5 logs higher than in parental wild-type cells or those lacking STING or TRIF (Fig. 6A). This suggests that the comparatively low replication of the virus in fibroblasts may be due to infection-induced, TRIF- and STING-independent but IPS-1/MAVS and IFN-dependent activity in this cell type (see below). ZIKV also exhibited enhanced growth in cells lacking IPS-1/MAVS and IFNAR, potentially indicating that this represents a flavivirus-specific trait. Importantly, these observations also establish IPS-1/MAVS-deficient cells as a permissive and useful model for assessing the role of TRIF or STING activation in DENV growth, including AV-C-mediated effects. Unfortunately, AV-C did not stimulate observable antiviral effects against CHIKV in C57BL/6J mice, as we have previously shown for the characterized IRF3 agonist DMXAA (5,6-dimethylxanthenone-4-acetic acid) (41) (Fig. S5). Given the requirement for functional IFN signaling, this was likely due to the fact that AV-C was unable to trigger IFN-stimulated gene transcription in murine cells *in vitro* or secretion of serum-associated type I IFN in mice, as also seen with DMXAA (Fig. S4).

10.1128/mBio.00452-17.5 FIG S5  *In vivo* activity of AV-C. (A) Transcription of IFN-inducible genes IP10, IFIT1, and ISG15 in murine RAW 264.7 macrophage-like cells treated for 8 h with universal type I IFN (uIFN) or 25 μM AV-C. Values displayed are average fold changes versus results in DMSO-treated cells from two biological replicates. (B) Transcription of IP10, IFIT1, and ISG15 in murine RAW 264.7 macrophage-like cells treated for 8 h with serum harvested 6 h posttreatment from C57BL/6J mice injected intraperitoneally with DMXAA or AV-C (25 mg/kg). Values displayed are average fold changes versus DMSO-treated cells of two biological replicates. (C) Average (± standard deviations) CHIKV titers from homogenates of the indicated tissues at 3 days postinfection from C57BL/6J mice (*n* = 4) treated intraperitoneally with DMSO or AV-C at 25 mg/kg. Download FIG S5, PDF file, 0.1 MB. Copyright © 2017 Pryke et al. 2017 Pryke et al. This content is distributed under the terms of the Creative Commons Attribution 4.0 International license.

### AV-C elicits innate immune reactivity in human peripheral blood mononuclear cells.

**FIG 7:**
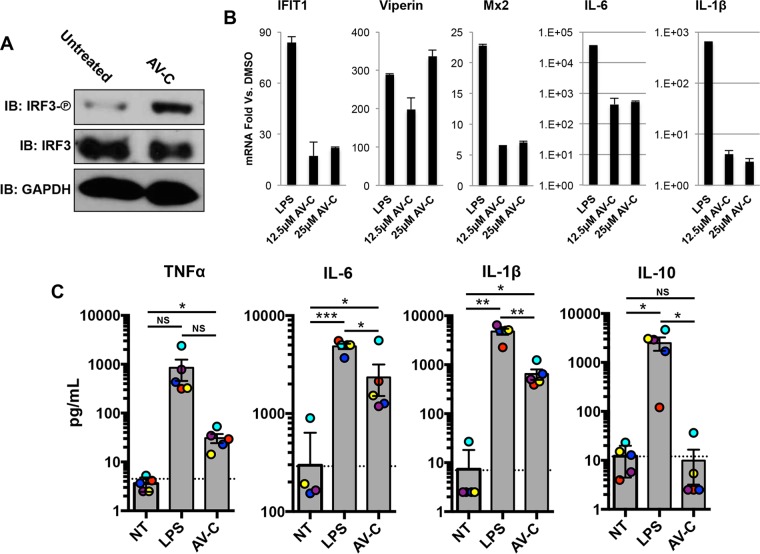
AV-C-mediated innate activation of primary human peripheral blood mononuclear cells. (A) Immunoblot showing the S386 phosphorylation status of IRF3, total IRF3, and GAPDH from human PBMC lysates following 4-h treatment with 1% DMSO or 12.5 μM AV-C. (B) Transcription of IFIT1, viperin, Mx2, IL-6, and IL-1β in PBMCs treated for 8 h with 1 μg/ml LPS, 12.5 μM AV-C, or 25 μM AV-C. Values displayed are average fold changes versus results in DMSO-treated cells of two biological replicates, ± standard deviations. (C) Secretion of TNF-α, IL-6, and IL-1β from PBMCs either left untreated (NT) or exposed for 24 h to 100 ng/ml LPS or 25 μM AV-C. Values presented are averages (in picograms per milliliter) ± the standard deviation from cells from five individual donors (donor-specific values are color coded). Paired-sample Student’s *t* test comparisons were made between treated and NT cells. *, *P* < 0.05; **, *P* < 0.01; ***, *P* < 0.001.

The ability of innate activating stimuli to induce secretion of immunomodulatory cytokines is strongly predictive of their capacity to initiate and facilitate adaptive immune responses (e.g., adjuvanticity). This is exemplified by the TLR4/TRIF agonist monophosphoryl lipid A (MPL), which is used clinically as a vaccine adjuvant (67). To investigate whether AV-C is capable of similarly inducing innate activation of primary cells relevant to the establishment of adaptive responses, we examined reactivity in human PBMCs. Observation of such activity would represent an important proof of concept for the potential expansion of AV-C’s therapeutic utility. As shown in Fig. 7A, AV-C stimulated IRF3 S386 phosphorylation in these cells. Furthermore, Fig. 7B illustrates that AV-C was able to induce transcription of endogenous ISGs in PBMCs (as observed in human fibroblasts) as well as proinflammatory genes IL-6 and IL-1β. Next, we examined the ability of AV-C to stimulate (i) the secretion of cytokines associated with potentiating adaptive immune responses to microbial pathogens following antigen exposure, as well as (ii) expression of cellular markers indicative of dendritic cell (DC) maturation. This was done using PBMCs from five separate human donors, to control for potentially complicating impacts of biological characteristics known to influence innate immune induction, such as genetic background or age. As shown in Fig. 7C, treatment of these cells for 24 h with AV-C was found to significantly induce secretion of the proinflammatory cytokines tumor necrosis factor alpha (TNF-α), IL-6, and IL-1β relative to levels in untreated cells from matched donors. Similarly trending results were observed for the TLR4/TRIF stimulus LPS. Interestingly, however, while LPS significantly induced secretion of the anti-inflammatory cytokine IL-10, the same was not true for AV-C. Furthermore, AV-C was also unable to upregulate expression of the maturation markers HLA-DR, CD86, CD80, or CD40 on immature DCs (Fig. S7). These observations suggest that both TRIF activators LPS and AV-C may fundamentally induce distinct cellular target pathways. Nevertheless, AV-C is capable of stimulating an IRF3-associated innate immune response in primary PBMCs from multiple human donors, thereby magnifying its potential as an immunotherapeutic compound.

### AV-C does not trigger canonical activation of NF-κB.

**FIG 8:**
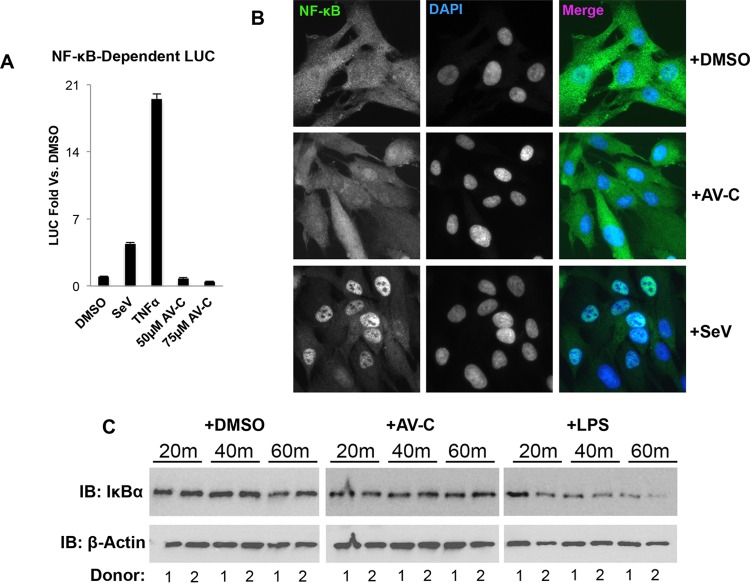
NF-κB activity is not induced by exposure to AV-C. (A) Reporter assay results, showing induction of NF-κB-dependent LUC expression in THF–NF-κB cells at 8 h posttreatment with 160 HAU/ml SeV, 10 ng/ml TNF-α, or the indicated concentration of AV-C. Values displayed are average fold changes (± standard deviations) based on three replicates compared to DMSO-treated cells. (B) Subcellular localization of NF-κB subunit P65 in THF cells following 4-h exposure to 1% DMSO, 160 HAU/ml SeV, or 25 μM AV-C. (C) Degradation of IκBα in PBMCs from two donors following exposure to 1% DMSO, 12.5 μM AV-C, or 100 ng/ml LPS for the indicated time (β-actin served as the loading control).

NF-κB is a transcription factor that is activated in response to signaling from multiple PRRs, many that also lead to IRF3, and is important for the expression of numerous proinflammatory cytokines (68), including type I IFNs (69). We therefore examined whether exposure of cells to AV-C led to responses indicative of NF-κB activation. As shown in Fig. 8A, THF cells stably transfected with NF-κB-dependent LUC (41) did not respond to AV-C at concentrations above those found to induce IFN-dependent signaling. We next used indirect immunofluorescence to examine the subcellular localization of NF-κB subunit P65 in THFs following AV-C treatment. Figure 8B illustrates that exposure to the established NF-κB-inducing stimulus SeV led to nuclear accumulation of P65, a requisite step in the protein’s transcriptional activity. However, nuclear accumulation of the protein was not detected in DMSO- or AV-C-exposed cells, consistent with NF-κB not being activated by these treatments. Intriguingly, NF-κB has been shown to play a role in transcription of cytokines, such as TNF-α, IL-6, and IL-1β, that we found to be secreted by PBMCs in response to AV-C (Fig. 7C). As such, we decided to ask whether the lack of AV-C-mediated NF-κB activation was a phenomenon specific to fibroblasts. To address this, we exposed PBMCs from two donors to AV-C at 25 μM, a concentration that elicited cytokine expression and secretion in these cells (Fig. 7). Control cells were treated with DMSO (negative) or LPS (positive). Whole-cell lysates were then harvested at 20 min, 40 min, and 60 min posttreatment, and immunoblotting was used to examine levels of IκBα, a factor that sequesters NF-κB in the cytoplasm and whose ubiquitin-mediated degradation is required for NF-κB nuclear accumulation. As shown in Fig. 8C, treatment of PBMCs with LPS led to substantial degradation of IκBα in both donor cells by 60 min. However, treatment with DMSO or AV-C failed to trigger degradation of the protein. Based on these observations, we concluded that AV-C does not induce cellular signaling responses that lead to activation of NF-κB in either fibroblasts or PBMCs.

## DISCUSSION

The ongoing transmission of multiple arboviruses in Latin America and the Caribbean underscores the value of developing therapeutic strategies that display broad-spectrum efficacy. Unfortunately, the fact that the current outbreak involves unrelated virus families (i.e., *Togaviridae*, *Flaviviridae*) that exhibit unique replication characteristics has made identification of multitarget, directly acting antiviral molecules more problematic. Fortunately, the type I IFN system, as part of the overall innate immune response, evolved to effectively block replication by diverse viral pathogens, including flaviviruses and alphaviruses (70, 71). Clinical utilization of IFN itself is, however, associated with significant undesirable attributes, including high cost, dosing feasibility, and adverse side effects (30, 31, 72). Nevertheless, the potency of IFN-induced processes renders them druggable yet underutilized therapeutic targets (33). Furthermore, the high probability of sensitivity of unknown yet potentially emerging viral pathogens (or bioweapons) to the IFN-induced cellular state provides another motivation for drug discovery endeavors with this focus. The ability of innate immune stimuli to yield other immunotherapeutic outcomes, such as adjuvanticity and antitumor activity, adds still further incentive to their discovery and characterization (73). Our group and others have pursued novel agonists of IRF3-terminal pathways as treatments against RNA viruses, including DENV (35–38, 74) and CHIKV (36, 37, 41). However, the current study represents the first application of this approach to ZIKV. Moreover, while molecules described thus far as efficacious are agonists of the IPS-1/MAVS or STING pathways, our study describes a novel synthetic compound that induces TRIF-dependent responses. Indeed, TRIF may represent a curiously underemphasized cellular target for combating DENV, ZIKV, and alphavirus infections. This is due to the fact that while virus-inhibitory phenotypes have been described that are directed at IPS-1/MAVS (75, 76) and STING (77), TRIF-targeting mechanisms have thus far not been identified in these viruses to our knowledge. This is supported by our observation that replication of DENV and ZIKV appears similar in wild-type cells and those lacking TRIF (Fig. 6). As such, TRIF-dependent activation of the IFN response could be a more potent antiviral strategy, since this pathway may be less vulnerable or even invulnerable to disruption by these viruses.

Initially, our work retrieved multiple small molecules capable of inducing IFN-dependent reporter signaling (see Fig. S1 in the supplemental material), and many of these are being actively characterized in our laboratory. AV-C was found to robustly generate consistent responses in multiple cell types, and we therefore concentrated our efforts on understanding its mode of action and therapeutic potential. We began by examining the cellular transcriptional response induced by the molecule and the similarities shared with that induced by type I IFN. While a larger set of genes regulated by IFN treatment was uncovered, a substantial subset was found to be co-upregulated by AV-C treatment, including many that confer direct antiviral activity, as well as IFN-β itself (Fig. 2B). Importantly, some of these (including IFN-β) were reflected in corresponding AV-C-induced protein translation (Fig. 2C and 6C), suggesting that despite upregulation of a limited group of IFN-stimulated genes, AV-C may generate a cellular environment antagonistic to virus growth. We therefore examined replication of CHIKV, DENV, and ZIKV on fibroblasts pretreated with AV-C to determine whether this effect was evident. As shown in Fig. 3, all three viruses grew to diminished levels in AV-C-pretreated THF cells, with CHIKV displaying the highest sensitivity to the compound. Interestingly, the overall impairment of ZIKV growth was less pronounced than that observed for CHIKV. This observation may be consistent with the encoding of phenotypes by ZIKV (such as a block to STAT2 function [78]) that enhance its resistance to IFN-induced cellular states. The extent to which viral mechanisms are involved in the resistance of ZIKV to IFN-stimulated cells will require a more focused deconstruction.

Activation of IFN and IFN-dependent genes can occur by way of multiple transcription factors and signaling pathways. To elucidate the cellular proteins involved in the AV-C-associated antiviral state under the hypothesis that IFN is involved, we examined specific components of the best-characterized IFN-terminal signaling cascades. IRFs, including IRF1, IRF5, IRF7, and IRF3, as well as NF-κB, have been implicated in the transcriptional induction of IFN genes (reviewed in reference 68). We first examined the activation status of IRF3 and found that, as with conventional stimuli of the STING, IPS-1/MAVS, and TRIF pathways, AV-C did in fact induce phosphorylation of the protein in fibroblasts (Fig. 4A). Whether IRF3 and its activation by AV-C are actually required for transcriptional induction were separate questions that we addressed by using cells that lacked the IRF3 protein as well as using a chemical inhibitor of TBK1/IKKε (BX795). In both of these experiments, AV-C failed to activate expression of an IFN-dependent reporter or endogenous mRNAs. Furthermore, AV-C-associated IRF3 phosphorylation was also abrogated in the presence of BX795 (Fig. 4). Collectively these results indicate that AV-C triggers an IRF3-terminal pathway, of which three primary PRR- and adaptor-associated ones are known. We therefore next focused on addressing whether any of these was necessary for the AV-C-induced innate activation phenotype.

An understanding of the target cellular pathway activated by AV-C represents critical information that allows wider predictions to be made about tissue states and phenotypes elicited by the molecule. For example, dsRNA can lead to disparate outcomes, including transcription-independent effects, such as apoptosis and Src-mediated cell migration, depending on whether it activates the RIG-I/IPS-1/MAVS- or TLR3/TRIF-dependent pathways (79). *In vivo*, the RIG-I and TLR3 ligands can also produce distinct adaptive immune response profiles to coadministered vaccine antigens (80, 81). Furthermore, secretion of dissimilar cytokine profiles is evident following STING-mediated versus TLR/TRIF-mediated stimulation (40, 82). In light of these findings, we aimed to identify the adaptor-associated signaling pathway(s) required for AV-C-induced IRF3 activation. We found that AV-C-induced transcription and IRF3 phosphorylation were operational in cells lacking either STING or IPS-1/MAVS (Fig. 5). In the absence of TRIF, however, innate stimulation by AV-C as defined by these activities was eliminated. Whether AV-C also triggers related adaptors, such as MyD88, or upstream PRRs, such as TLR3 or TLR4, is currently being examined. Recent work has uncovered a functional role for TRIF in STING-dependent innate activation (83). Our data reveal no obvious differences in AV-C-mediated innate induction or biological activity between wild-type cells and those lacking STING (Fig. 5 and 6), suggesting that the compound activates a TRIF-specific pathway independently of STING.

AV-C treatment leads to TRIF-dependent transcription of IFN-β (Fig. 2) as well as antiviral ISGs that are known to inhibit flavivirus and alphavirus replication (84–86). We therefore asked whether the TRIF, IPS-1/MAVS, and STING signaling pathways are necessary for the AV-C-associated antiviral phenotypes we observed (Fig. 3). As shown in Fig. 6, cell lines lacking each individual protein were able to establish canonical IFN-induced antiviral states as indicated by the strong inhibition of all three viruses following pretreatment with IFN-β, indicating that JAK/STAT signaling is functional. AV-C was also able to induce equivalent (and perhaps more potent) antiviral activity in cells lacking IPS-1/MAVS and STING, indicating that these proteins are dispensable for antiviral effects conferred by AV-C, in agreement with results presented in Fig. 5. Interestingly, AV-C induced the expression of antiviral genes, such as viperin, IFIT1, and ISG15, that can be transcribed in an IRF3-dependent, IFN-independent manner (65, 66). Moreover, growth of all three viruses was highly susceptible to cellular states elicited by IFN-β pretreatment (Fig. 6A). We therefore examined whether IFN-induced (JAK/STAT-mediated) signaling was, along with TRIF signaling, a mechanistic requirement for the antiviral state associated with AV-C exposure. To address this, we constructed cells from which the IFNAR protein was deleted and were correspondingly unable to react to type I IFN, as demonstrated by the failure to establish an IFN-induced antiviral state. As shown in Fig. 6A, the antiviral effect of AV-C was also abrogated in these cells, suggesting that AV-C-induced, TRIF/IRF3-dependent secretion of type I IFN is responsible for the compound’s activity. To further validate this conclusion, we examined whether cells exposed to AV-C actually secreted bioactive type I IFN in detectable quantities. We first utilized a cell-based bioassay to show that AV-C-treated wild-type THF cells as well as those lacking IPS-1/MAVS or STING, but not TRIF, secreted type I IFN (Fig. 6B). Additionally, human PBMCs exposed to AV-C secreted IFN-β, demonstrating that multiple cell types respond in this way (Fig. 6C). Collectively, these results are consistent with a model of AV-C-mediated activity that involves TRIF-dependent but STING- and IPS-1/MAVS-independent synthesis and secretion of type I IFNs that generate an antiviral cellular state through ISG expression following engagement of the IFNAR and ensuing JAK/STAT signaling.

Unfortunately, wild-type fibroblasts represent a less useful substrate for growth of DENV. Our experiments revealed, intriguingly, that this is likely due to the virus’ inability to counteract infection-induced, IFN-mediated antiviral responses in these cells. DENV growth was enhanced by nearly 2 logs in THFs engineered to lack either IPS-1/MAVS or IFNAR (Fig. 6A) (87, 88). These results align well with the observation that DENV is extremely sensitive to the IFN-induced state in fibroblasts (Fig. 6A), as well as previous reported findings showing that the IPS-1/MAVS/IRF3 signaling axis is crucial to flavivirus-associated IFN induction (76, 89, 90). Importantly, this also indicates that THF-ISRE-ΔIPS1 cells represent a sound model for examining the antiviral effect of AV-C since (i) they are capable of responding to AV-C-induced, IRF3-terminal signaling (Fig. 5), (ii) their IFN-induced signaling is intact, and (iii) they support high-titer DENV growth. Enhanced replication of ZIKV, a related flavivirus, was also observed in the absence of either IPS-1/MAVS or IFNAR, indicating that it may, as expected, trigger IPS-1/MAVS-dependent IFN synthesis. Replication of CHIKV did not differ significantly between the mutant cell lines, despite the fact that the virus is highly sensitive to the IFN-induced phenotype (Fig. 6A). This may have been due to the nonspecific nature of the virus’ innate evasion strategy, which involves rapid and indiscriminate shutdown of cellular gene transcription and translation (56). Whether CHIKV exhibits IRF3-terminal pathway-targeted inhibition has not been demonstrated to our knowledge.

TRIF-inducing stimuli may possess immunotherapeutic potential beyond direct antiviral activity (91–93). For instance, the TLR3/TRIF agonist poly(I-C) shows beneficial effects in mouse models of arthritis (94), colitis (95), bacterial infection (96), and wound healing (97, 98). Likely more impactful are the potentiating effects of TRIF stimulation on establishment of adaptive immunity to infectious agents, as illustrated by the TLR4/TRIF agonist MPLA, an FDA-approved component of vaccines against papillomavirus (99) and hepatitis B virus (67). The fundamental mechanistic basis for the adjuvant activity conferred by MPLA is TRIF- and MyD88-mediated synthesis of proinflammatory cytokines and chemokines that drive recruitment and maturation of myeloid-derived antigen-presenting cells (100–102). Ultimately, this leads to an enhancement of antigen-associated T cell activation, clonal expansion, and Th1 polarization (67, 103). To investigate whether AV-C can induce cellular responses predictive of these immune effects, we examined both secretion of relevant cytokines from primary PBMCs collected from human patients as well as expression of cellular markers that signify DC maturation. As illustrated in Fig. 7, AV-C induced both IRF3 phosphorylation in PBMCs as well as transcription of IFN-stimulated genes IFIT1, viperin, and Mx2. The compound also triggered secretion of key proinflammatory molecules TNF-α, IL-6, and IL-1β (Fig. 7C), suggesting that it may elicit responses contributory to increased antigen immunogenicity. Interestingly, the innate reactivity to AV-C diverged qualitatively from that induced by the TLR4/TRIF agonist LPS. Transcription of IL-6 and IL-1β was strongly activated by LPS, but much less so by AV-C (Fig. 8B), and while LPS led to secretion of IL-10, AV-C did not (Fig. 8C). Furthermore, AV-C also failed to stimulate expression of DC maturation markers HLA-DR, CD86, CD80, and CD40 on immature DCs and did not elicit secretion of IL-12p70 from these cells (Fig. S6). These differences underscore the need for more thorough characterization of the molecule in the context of adaptive immune processes to more completely evaluate its potential as an immunotherapeutic agent.

10.1128/mBio.00452-17.6 FIG S6  AV-C does not induce maturation of myeloid dendritic cells or secretion of IL-12p70. (A) Myeloid dendritic cells differentiated from healthy human PBMCs were treated with 1% DMSO or stimulated with 0.5 μg/ml LPS plus 40 ng/ml IFN-γ and 12.5 µM or 25 µM AV-C for 20 h. DCs were harvested and analyzed by flow cytometry for the upregulation of surface HLA-DR as well as the costimulatory molecules CD86, CD80, and CD40. Values presented are average mean fluorescence intensities (MFI) ± the standard deviations for the indicated marker from five individual donors (donor-specific values are represented by clear circles). (B) Myeloid dendritic cells differentiated from healthy human PBMCs were treated as described for cells shown in panel A for 20 h. Culture supernatants were analyzed for the level of IL-12p70 via an ELISA. Values presented are average picograms per milliliter ± the standard deviation from cells of four individual donors (donor-specific values are represented by clear circles). Paired-sample Student’s *t* test comparisons were made between DMSO-treated and stimulated cells. ns, nonsignificant; *, *P* < 0.05; **, *P* < 0.01; ***, *P* < 0.001. Download FIG S6, PDF file, 0.7 MB. Copyright © 2017 Pryke et al. 2017 Pryke et al. This content is distributed under the terms of the Creative Commons Attribution 4.0 International license.

The induction of proinflammatory cytokines such as TNF-α, IL-6, and IL-1β by AV-C suggests the involvement of signaling pathways or transcription factors beyond IRF3 alone. Unexpectedly, AV-C failed to induce outcomes such as secretion of IL-10 and IL-12p70 that were induced by TLR4/TRIF agonist LPS (Fig. 7; Fig. S6). These results may represent differential impacts of MyD88 in associated signaling induced by the two stimuli, since secretion of some proinflammatory cytokines, such as IL-10, is known to be abrogated in cells lacking MyD88 (104–106). Based on the reported role of NF-κB in transcription of proinflammatory genes such as TNF-α, IL-6, IFN-β, and IL-1β, which are induced by AV-C as well, as well as dependence on TRIF and MyD88 signaling, we decided to examine whether the compound led to activation of this factor. Surprisingly, we were unable to detect indicators of AV-C-induced NF-κB activation in either THFs or PBMCs (Fig. 8). These results may be suggestive of the absence of involvement of MyD88 in the AV-C-stimulated response, since NF-κB induction represents a process enhanced by MyD88 rather than TRIF activity (60, 107). This is also consistent with the differential induction of IL-10 by AV-C and LPS, the expression of which has been linked to NF-κB activity in immune and other cells (108–110). With regard to the co-upregulation of TNF-α, IL-6, and IL-1β by AV-C and LPS, NF-κB-independent roles for other transcription factors, such as IRF1 and IRF5, in the expression of such proinflammatory cytokines have been described (111, 112). Whether AV-C is capable of activating other IRFs such as these is certainly possible but requires specific examination.

An assessment of the clinical utility of AV-C would be greatly enabled by a reactive animal model in which the scope of physiological responses stimulated by the compound could be measured. This includes evaluation of the impact of AV-C on adaptive immunity, the molecule’s *in vivo* antiviral efficacy, systemic inflammatory responses, and immunopathology. The lack of innate reactivity observed in mice or in murine cells (Fig. S5) may be due to myriad causes whose characterization is unfortunately beyond the scope of this work. However, in an attempt to permit *in vivo* experimentation, we constructed multiple chemical analogs of AV-C with the goal of finding one or more that induces innate responses in both human and mouse cells. As illustrated in Fig. S7, even slight chemical modifications of AV-C rendered it inactive in human cells (e.g., compare AV-C, AV-C-09, and AV-C-10). As such, we suspect that the molecule’s stimulatory capacity is extremely sensitive to structural alteration, perhaps due to a protein interaction involving small or precisely shaped regions. We are, however, examining whether the molecule activates cells from species more phylogenetically similar to humans, such as rhesus or Japanese macaques. In the event that IRF3 and IFN are induced by the compound, these may represent tractable animal models for *in vivo* characterization. While more complex and expensive than murine models, nonhuman primates offer the ability to obtain information about *in vivo* activities that more closely resemble those expected in humans.

10.1128/mBio.00452-17.7 FIG S7  Innate induction by AV-C analogs. Luminescence from THF-ISRE cells following 8 h of exposure to multiple concentrations of the indicated AV-C derivative molecules. Download FIG S7, PDF file, 0.2 MB. Copyright © 2017 Pryke et al. 2017 Pryke et al. This content is distributed under the terms of the Creative Commons Attribution 4.0 International license.

## MATERIALS AND METHODS

### Reagents and antibodies.

Dimethyl sulfoxide was obtained from Thermo Fisher. Puromycin was obtained from Clontech and used at 3 μg/ml in cell culture medium. LPS and Polybrene were obtained from Sigma-Aldrich. Human recombinant IFN-β, IFN-γ, universal mammalian IFN, and TNF-α were obtained from PBL. Granulocyte-macrophage colony-stimulating factor (GM-CSF) and IL-4 were obtained from Gemini Bio-Products. Steady-Glo cell lysis/luciferin reagent was obtained from Promega. Poly(I-C) was obtained from GE Healthcare. Stocks of G10 were synthesized by the OHSU Medicinal Chemistry Core Facility. DMXAA was purchased from ApexBio. Antibodies against the following antigens were used (with the source indicated in parentheses): glyceraldehyde-3-phosphate dehydrogenase (GAPDH; sc-51906; Santa Cruz Biotechnology); IRF3 (sc-9082; Santa Cruz Biotechnology); human S386 phospho-IRF3 (catalog number 2562-1; Epitomics); STING (catalog number 3337; Cell Signaling); IPS-1 (A300-782A; Bethyl); IFIT1/ISG56 (PA3 848; Thermo Fisher); TRIF (4596; Cell Signaling); Mx1 (GTX111153; GeneTex); β-actin (MAB0501R; Thermo Fisher); IκBα (Santa Cruz sc-371; Santa Cruz Biotechnology); NF-κB P65 (sc-372; Santa Cruz Biotechnology).

### Cell and virus culture.

Human foreskin fibroblasts originally obtained from the American Type Culture Collection (ATCC) were stably transduced with constitutively expressed human telomerase reverse transcriptase and the IRF3/IFN-responsive pGreenFire-ISRE lentivector and were maintained in Dulbecco’s modified Eagle’s medium (DMEM) containing 10% fetal calf serum (FCS) and antibiotics as described previously (45). Vero, BHK-21, and C6/36 cells were obtained from ATCC and were grown as described elsewhere (56). RAW 264.7 and THP-1 cells were obtained from Jay Nelson (Oregon Health and Science University). THP-1 cells were differentiated in 100 nM phorbol 12-myristate 13-acetate (PMA) for 24 h before stimulation. Human peripheral blood mononuclear cells were obtained either from iXcells Biotechnologies or from healthy donors (Providence IRB PHS 13-026A; Drexel University IRB 1506003707A005) and maintained in RPMI containing 10% FCS and antibiotics. Human umbilical microvascular endothelial cells were obtained from Ashlee Moses (Oregon Health and Science University) and maintained as described previously (113). All cells were grown at 37°C and 5% CO_2_. Sendai virus was obtained from Charles River Laboratories, Inc. and used at 160 hemagglutinating units (HAU)/ml. Cytomegalovirus was grown, virus titers were determined, the virus was UV inactivated, and cells were exposed to virus as described previously (61). Dengue virus serotype 2 was used as previously described (114). Zika virus strain PRVABC59 was obtained from and isolated by the Centers for Disease Control and Prevention (CDC) from an individual in Puerto Rico in December 2015 (115). The virus was passed twice on C6/36 cells, and working stock was concentrated through a 20% sorbitol cushion. CHIKV strain MH56 was obtained from Michael Diamond (Washington University). CHIKV was derived from an infectious clone as follows. RNA was transcribed from the linearized clone using the T7 mMessage mMachine kit (Ambion) and transfected via Lipofectamine into BHK-21 cells. Resultant virus was propagated in C6/36 insect cells for 48 h to produce high-titer viral stocks after pelleting through a 20% sucrose cushion by ultracentrifugation (22,000 rpm [825,206 × *g*] for 1.5 h). Experimental infections were carried out in triplicate using a multiplicity of infection (MOI) of 1 PFU per cell for CHIKV and 5 PFU per cell for DENV and ZIKV. Virus was quantified by serial dilution on Vero cells on a carboxymethylcellulose overlay. CHIKV was measured in a cytopathic effect (CPE)-based assay, and DENV and ZIKV were measured in focus-forming unit (FFU) assays (116). Cell viability was examined by quantitating ATP via the Cell Titer Glo assay (Promega) according to the manufacturer’s instructions.

### Lentivector transduction and CRISPR/Cas9-mediated genome editing.

Genome editing using lentivector-mediated delivery of CRISPR/Cas9 components was performed generally as described previously (41, 117). Briefly, 20-nucleotide guide RNA (gRNA) sequences targeting protein-coding regions were inserted into the lentiCRISPRv2 vector (AddGene 52961). These sequences were as follows. TRIF, CCTAGCGCCTTCGACATTCT; IFNAR, AAACACTTCTTCATGGTATG; IRF3, GAGGTGACAGCCTTCTACCG; STING, CCCGTGTCCCAGGGGTCACG; IPS-1, AGTACTTCATTGCGGCACTG. Sequence verification of genomic disruption for each cell line is presented in Fig.  8. Lentivirus was made by transfecting specific lentiCRISPRv2 plasmid along with packaging plasmid (psPAX2; AddGene 12260) and vesicular stomatitis virus G protein pseudotyping plasmid (pMD2.G; Addgene 12259) into Lenti-X 293T cells (Clontech) using Lipofectamine-LTX (Life Technologies, Inc.). Medium was harvested at 48 h and 72 h posttransfection, centrifuged (3,000 × *g* for 10 min), and filtered through a 0.45-μm filter to remove cell debris. Subconfluent target cells were exposed to lentivirus for 8 h in the presence of 5 μg/ml Polybrene. After the cells reached confluence, cultures were split into DMEM plus 10% FCS containing 3 μg/ml puromycin. Transduced cells were passaged in the presence of puromycin for 7 to 10 days before protein knockout was examined by immunoblotting. Cells were next serially diluted twice in 96-well plates to obtain oligoclonal lines purified for gene deletion. Sanger sequence verification of genomic disruption for each cell line is presented in Fig. S8. Protein knockout was additionally verified functionally by measuring phenotypic responsiveness to appropriate stimuli.

10.1128/mBio.00452-17.8 FIG S8  CRISPR/Cas9-mediated disruption of coding regions. The Sanger sequencing electropherograms show genomic regions near gRNA targeting sites of the indicated protein coding region along with corresponding gRNA sequences. Download FIG S8, PDF file, 0.3 MB. Copyright © 2017 Pryke et al. 2017 Pryke et al. This content is distributed under the terms of the Creative Commons Attribution 4.0 International license.

### Luciferase reporter and type I IFN bioassays.

For direct THF-ISRE reporter assays, confluent cells were plated at 20,000 cells per well in a white 96-well plate 24 h before stimulation. Treatments were performed in quadruplicate in 50 μl DMEM plus 2% FCS for 8 h unless otherwise indicated. Steady-GLO lysis/luciferin reagent (Promega) was added 1:1 to each well, and luminescence was measured on a Synergy plate reader (BioTek). For type I IFN bioassays, cells of interest were plated at 50,000/well in 24-well plates and serum starved in X-Vivo15 medium for 1 h prior to treatment. After being treated for 24 h, treated medium was harvested and clarified at 10,000 × *g* for 3 min. Recombinant IFN-β was used as the standard at 40, 20, 10, 5, 2.5, 1.25, and 0.63 U/ml. Medium was then added to THF-ISRE-ΔIRF3 cells plated as described above for 8 h, and luminescence was measured. IFN was quantitated by curve fitting relative to signals generated from standards.

### Affymetrix microarray hybridization and analysis.

Microarray assays were performed in the OHSU Gene Profiling Shared Resource. RNA sample quantity and purity were measured by UV absorbance at 260, 280, and 230 nm with a NanoDrop 1000 spectrophotometer (ThermoScientific). RNA integrity and size distribution were determined by running 200 ng of each sample on an RNA 6000 Nano chip instrument (Agilent Technologies). Total RNA samples were prepared for array hybridization by labeling 100-ng aliquots using the 3′IVT Express kit (Affymetrix). RNA was reverse transcribed to generate first-strand cDNA containing a T7 promoter sequence. A second-strand cDNA synthesis step was performed that converted the single-stranded cDNA into a dsDNA template for transcription. Amplified and biotin-labeled cRNA was generated during the *in vitro* transcription step. After a magnetic bead purification step to remove enzymes, salts, and unincorporated nucleotides, the cRNA was fragmented. According to standard Affymetrix recommendations, the labeled and fragmented cRNA was combined with hybridization cocktail components and hybridization controls, and 130 μl of each hybridization cocktail containing 6.5 µg of labeled target was injected into a cartridge containing the GeneChip Human Primeview array (Affymetrix). This array contains 49,395 probe sets corresponding to content derived from the RefSeq and UniGene databases. Arrays were incubated for 18 h at 45°C, followed by washing and staining on a GeneChip Fluidics Station 450 (Affymetrix) and the associated hybridization wash and stain kit (Affymetrix). Arrays were scanned using the GeneChip Scanner 3000 7G with an autoloader (Affymetrix). Image inspection was performed manually immediately following each scan. Image processing of sample .DAT files to generate probe intensity .CEL files was performed using the Affymetrix GeneChip Command Console (AGCC) software. Each array file was then analyzed using Expression Console v. 1.1, software (Affymetrix) to generate array performance metrics on nonnormalized data. Array performance and general data quality were assessed using the following values: background intensity, PM (perfect match) mean, Probeset mean, MAD (median absolute deviation) residual mean, RLE (relative log expression) mean, and housekeeping control probes (actin and GAPDH). All arrays passed GPSR performance-quality thresholds. To identify probe sets that were significantly regulated in treated versus untreated (mock) cells, we employed a traditional unpaired one-way (single-factor) ANOVA for each pair of condition groups as implemented in the Transcriptome Analysis Console (TAC) software. Probe sets were considered differentially regulated if the ANOVA *P* value was <0.05.

### Immunoblotting.

Sodium dodecyl sulfate-polyacrylamide gel electrophoresis (SDS-PAGE) immunoblotting was performed as follows. After trypsinization and cell pelleting at 2,000 × *g* for 10 min, whole-cell lysates were harvested in RIPA lysis buffer (50 mM Tris-HCl [pH 8.0], 150 mM NaCl, 1% NP-40, 0.5% sodium deoxycholate, and 0.1% SDS) supplemented with Halt protease and phosphatase inhibitor cocktail (Thermo Fisher). Lysates were electrophoresed in 8% polyacrylamide gels and transferred onto polyvinylidene difluoride membranes (Millipore) using semidry transfer at 400 mA for 1 h. The blots were blocked at room temperature for 2 h or overnight using 10% nonfat milk in 1× phosphate-buffered saline (PBS) containing 0.1% Tween 20. The blots were exposed to primary antibody in 5% nonfat milk in 1× PBS containing 0.1% Tween 20 for 18 h at 4°C. The blots were then washed in 1× PBS containing 0.1% Tween 20 for 20, 15, and 5 min, followed by deionized water for 5 min. A 1-h exposure to horseradish peroxidase-conjugated secondary antibodies and subsequent washes were performed as described for the primary antibodies. The antibody was visualized using enhanced chemiluminescence (Pierce).

### Indirect immunofluorescence assay.

For the indirect immunofluorescence assay, cells were grown on coverslips in 24-well plates and treated as described above. At room temperature, cells were washed twice with PBS, fixed for 30 min in 3.7% formalin, washed, and quenched for 10 min using 50 mM NH_4_Cl. Cells were permeabilized with 0.1% Triton X-100 for 7 min and washed three times with PBS containing 2% bovine serum albumin (BSA). Cells were incubated with primary antibody in PBS containing 2% BSA at 37°C for 1 h, washed three times in PBS containing 2% BSA (10 min for each wash), and incubated with fluorescently conjugated secondary antibody diluted 1:1,000 in PBS containing 2% BSA for 1 h. Cells were washed twice in PBS containing 2% BSA (10 min for each wash) and once in PBS. Coverslips were mounted on a microscope slide with Vectashield mounting medium (Vector Laboratories, Burlingame, CA) containing 4′,6-diamidino-2-phenylindole, and imaging was performed on an Evos cell-imaging system.

### RNA isolation and semiquantitative reverse transcription-PCR.

Total RNA was isolated from cells, treated with DNase provided in a DNA-free RNA isolation kit (Zymo Research) according to the manufacturer’s protocol, and quantified by using UV spectrometry. Single-stranded cDNA for use as a PCR template was made from total RNA and random hexamers to prime first-strand synthesis via SuperScript III reverse transcriptase (Life Technologies, Inc.) per the manufacturer’s protocol. Comparison of mRNA expression levels between samples was performed using semiquantitative real-time RT-PCR (qPCR) with the Applied Biosystems sequence detection system according to the ΔΔ*C*_*T*_ method (118) with GAPDH as a control. Prevalidated Prime-Time 6-carboxyfluorescein qPCR primer/probe sets obtained from IDT were used for all genes.

### Cytokine analysis.

Human peripheral blood mononuclear cells were obtained from healthy donors and maintained as described above. Cells were plated at 4 × 10^5^ per well in 96-well plates, stimulated with DMSO, AV-C, or LPS (100 ng/ml) diluted in RPMI, and incubated at 37°C and 5% CO_2_ for 24 h. Supernatants were then removed and used in a multiplex cytokine bead-based assay according to the manufacturer’s protocol (BD Biosciences human inflammation cytokine bead array, catalog number 551811, or BioLegend human IL-12p70 enzyme-linked immunosorbent assay [ELISA] Max).

### Human myeloid DC differentiation from PBMCs.

PBMCs from healthy human control subjects were enriched for CD14^+^ monocytes by negatively selecting using the EasySep human monocyte enrichment kit without CD16 depletion (StemCell Technologies, Vancouver, Canada), according to the manufacturer’s protocol. Cells were then grown at a density of 2 × 10^6^/ml in serum-free Cellgro medium (RPMI 1640 1× plus l-glutamine) supplemented with human GM-CSF at 100 ng/ml and IL-4 at 20 ng/ml in 24-well plates. On day 3, differentiated DCs were stimulated with the indicated concentrations of AV-C for 20 h. LPS (0.5 μg/ml) plus human IFN-γ (40 ng/ml) was used as a positive-control stimulation for DC activation, and DMSO was used as the negative control.

### Flow cytometric analysis of AV-C-stimulated human DCs.

DCs were harvested 20 h poststimulation by pipetting the cells up and down. Cell death was assessed using the LIVE/DEAD fixable aqua dead cell stain kit (Life Technologies, Inc., Carlsbad, CA) as well as annexin V (BD Biosciences, San Jose, CA). Fc receptor blocking was performed with human TruStain FcX (BioLegend, San Diego, CA). DCs were stained with fluorochrome-conjugated antibodies against surface markers for 20 min on ice. The antibodies used were directed against CD3 (catalog number HIT3α), CD11c (3.9), HLA-DR (L243), CD86 (IT2.2), CD83 (HB15e), CD80 (2D10), and CD40 (5C3) and were all purchased from BioLegend (San Diego, CA). Anti-CD14 (M5E2) was obtained from eBioscience (San Diego, CA), and anti-CD19 (HIB19) was from BD Biosciences (San Jose, CA). DCs were then washed, samples were acquired on a BD LSR II apparatus, and data were analyzed using FlowJo software (version 9.9.3). DCs analyzed for costimulatory molecule expression were gated on the live, CD3/CD19-negative, CD11c-positive population.

### *In vivo* administration of AV-C and viral infection.

All animal procedures for in vivo administration of AV-C were conducted in accordance with and approved by the Oregon Health and Science University Institutional Animal Care and Use Committee (IACUC) under protocol 913. The Oregon Health and Science University IACUC adheres to the NIH Office of Laboratory Animal Welfare standards (OLAW welfare assurance A3304-1). C57BL/6J mice (5 to 7 weeks of age; Jackson Laboratories) were housed in cage units in an animal biosafety level 3 facility, fed *ad libitum*, and cared for under USDA guidelines for laboratory animals. AV-C at 24 mg/kg of body weight or DMXAA (or DMSO alone) was prepared in 50 μl DMSO and injected intraperitoneally. Mice were either challenged with 1,000 PFU CHIKV via footpad injection in 20 μl RPMI under isoflurane-induced anesthesia or euthanized at 6 h posttreatment for serum analysis. CHIKV-infected animals were euthanized at 72 h postinfection by isoflurane overdose. Blood was collected by cardiac puncture, and serum viral load titers were determined on Vero cells in duplicate as described above.

## References

[1] Musso D, Cao-Lormeau VM, Gubler DJ 2015 Zika virus: following the path of dengue and Chikungunya? Lancet 386:243–244. doi:10.1016/S0140-6736(15)61273-9. 26194519

[2] Brathwaite DO, San Martín JL, Montoya RH, del Diego J, Zambrano B, Dayan GH 2012 The history of dengue outbreaks in the Americas. Am J Trop Med Hyg 87:584–593. doi:10.4269/ajtmh.2012.11-0770. 23042846 PMC3516305

[3] Rodriguez-Morales AJ, Villamil-Gómez WE, Franco-Paredes C 2016 The arboviral burden of disease caused by co-circulation and co-infection of dengue, Chikungunya and Zika in the Americas. Travel Med Infect Dis 14:177–179. doi:10.1016/j.tmaid.2016.05.004. 27224471

[4] Roth A, Mercier A, Lepers C, Hoy D, Duituturaga S, Benyon E, Guillaumot L, Souares Y 2014 Concurrent outbreaks of dengue, Chikungunya and Zika virus infections—an unprecedented epidemic wave of mosquito-borne viruses in the Pacific 2012–2014. Euro Surveill 19:pii=20929. doi:10.2807/1560-7917.ES2014.19.41.20929. 25345518

[5] Long D, Long B, Koyfman A 2016 Zika virus: what do emergency physicians need to know? J Emerg Med 50:832–838. doi:10.1016/j.jemermed.2016.03.033. 27157106

[6] Villamil-Gómez WE, González-Camargo O, Rodriguez-Ayubi J, Zapata-Serpa D, Rodriguez-Morales AJ 2016 Dengue, Chikungunya and Zika co-infection in a patient from Colombia. J Infect Public Health 9:684–686. doi:10.1016/j.jiph.2015.12.002. 26754201

[7] Van Bortel W, Dorleans F, Rosine J, Blateau A, Rousseau D, Matheus S, Leparc-Goffart I, Flusin O, Prat C, Césaire R, Najioullah F, Ardillon V, Balleydier E, Carvalho L, Lemaître A, Noël H, Servas V, Six C, Zurbaran M, Léon L, Guinard A, van den Kerkhof J, Henry M, Fanoy E, Braks M, Reimerink J, Swaan C, Georges R, Brooks L, Freedman J, Sudre B, Zeller H 2014 Chikungunya outbreak in the Caribbean region, December 2013 to March 2014, and the significance for Europe. Euro Surveill 19:pii=20759. doi:10.2807/1560-7917.ES2014.19.13.20759. 24721539

[8] Christofferson RC 2016 Zika virus emergence and expansion: lessons learned from dengue and Chikungunya may not provide all the answers. Am J Trop Med Hyg 95:15–18. doi:10.4269/ajtmh.15-0866. PMC4944680 26903610

[9] Couderc TRS, Lecuit M 2015 Chikungunya virus pathogenesis: from bedside to bench. Antiviral Res 121:120–131. doi:10.1016/j.antiviral.2015.07.002. 26159730

[10] Samarasekera U, Triunfol M 2016 Concern over Zika virus grips the world. Lancet 387:521–524. doi:10.1016/S0140-6736(16)00257-9. 26852261

[11] Musso D, Gubler DJ 2016 Zika virus. Clin Microbiol Rev 29:487–524. doi:10.1128/CMR.00072-15. 27029595 PMC4861986

[12] Beckham JD, Pastula DM, Massey A, Tyler KL 2016 Zika virus as an emerging global pathogen: neurological complications of Zika virus. JAMA Neurol 73:875–879. doi:10.1001/jamaneurol.2016.0800. 27183312 PMC5087605

[13] Tang BL 2016 Zika virus as a causative agent for primary microencephaly: the evidence so far. Arch Microbiol 198:595–601. doi:10.1007/s00203-016-1268-7. 27412681

[14] tenOever BR 2016 The evolution of antiviral defense systems. Cell Host Microbe 19:142–149. doi:10.1016/j.chom.2016.01.006. 26867173

[15] Schoggins JW, Rice CM 2011 Interferon-stimulated genes and their antiviral effector functions. Curr Opin Virol 1:519–525. doi:10.1016/j.coviro.2011.10.008. 22328912 PMC3274382

[16] Thompson MR, Kaminski JJ, Kurt-Jones EA, Fitzgerald KA 2011 Pattern recognition receptors and the innate immune response to viral infection. Viruses 3:920–940. doi:10.3390/v3060920. 21994762 PMC3186011

[17] Meylan E, Curran J, Hofmann K, Moradpour D, Binder M, Bartenschlager R, Tschopp J 2005 Cardif is an adaptor protein in the RIG-I antiviral pathway and is targeted by hepatitis C virus. Nature 437:1167–1172. doi:10.1038/nature04193. 16177806

[18] Kawai T, Takahashi K, Sato S, Coban C, Kumar H, Kato H, Ishii KJ, Takeuchi O, Akira S 2005 IPS-1, an adaptor triggering RIG-I- and Mda5-mediated type I interferon induction. Nat Immunol 6:981–988. doi:10.1038/ni1243. 16127453

[19] Xu LG, Wang YY, Han KJ, Li LY, Zhai Z, Shu HB 2005 VISA is an adapter protein required for virus-triggered IFN-beta signaling. Mol Cell 19:727–740. doi:10.1016/j.molcel.2005.08.014. 16153868

[20] Seth RB, Sun L, Ea CK, Chen ZJ 2005 Identification and characterization of MAVS, a mitochondrial antiviral signaling protein that activates NF-κB and IRF3. Cell 122:669–682. doi:10.1016/j.cell.2005.08.012. 16125763

[21] Burdette DL, Monroe KM, Sotelo-Troha K, Iwig JS, Eckert B, Hyodo M, Hayakawa Y, Vance RE 2012 STING is a direct innate immune sensor of cyclic di-GMP. Nature 478:515–518. doi:10.1038/nature10429. PMC3203314 21947006

[22] Ishikawa H, Ma Z, Barber GN 2009 STING regulates intracellular DNA-mediated, type I interferon-dependent innate immunity. Nature 461:788–792. doi:10.1038/nature08476. 19776740 PMC4664154

[23] Sun W, Li Y, Chen L, Chen H, You F, Zhou X, Zhou Y, Zhai Z, Chen D, Jiang Z 2009 Eris, an endoplasmic reticulum IFN stimulator, activates innate immune signaling through dimerization. Proc Natl Acad Sci U S A 106:8653–8658. doi:10.1073/pnas.0900850106. 19433799 PMC2689030

[24] Zhong B, Yang Y, Li S, Wang YY, Li Y, Diao F, Lei C, He X, Zhang L, Tien P, Shu HB 2008 The adaptor protein MITA links virus-sensing receptors to IRF3 transcription factor activation. Immunity 29:538–550. doi:10.1016/j.immuni.2008.09.003. 18818105

[25] Ishikawa H, Barber GN 2008 STING is an endoplasmic reticulum adaptor that facilitates innate immune signalling. Nature 455:674–678. doi:10.1038/nature07317. 18724357 PMC2804933

[26] Oshiumi H, Matsumoto M, Funami K, Akazawa T, Seya T 2003 TICAM-1, an adaptor molecule that participates in toll-like receptor 3-mediated interferon-beta induction. Nat Immunol 4:161–167. doi:10.1038/ni886. 12539043

[27] Yamamoto M, Sato S, Mori K, Hoshino K, Takeuchi O, Takeda K, Akira S 2002 Cutting edge: a novel Toll/IL-1 receptor domain-containing adapter that preferentially activates the IFN-beta promoter in the toll-like receptor signaling. J Immunol 169:6668–6672. doi:10.4049/jimmunol.169.12.6668. 12471095

[28] Konerman MA, Lok AS 2016 Interferon treatment for hepatitis B. Clin Liver Dis 20:645–665. doi:10.1016/j.cld.2016.06.002. 27742005

[29] Ghany MG, Strader DB, Thomas DL, Seeff LB, American Association for the Study of Liver Diseases 2009 Diagnosis, management, and treatment of hepatitis C: an update. Hepatology 49:1335–1374. doi:10.1002/hep.22759. 19330875 PMC7477893

[30] Lin FC, Young HA 2014 Interferons: success in anti-viral immunotherapy. Cytokine Growth Factor Rev 25:369–376. doi:10.1016/j.cytogfr.2014.07.015. 25156421 PMC4182113

[31] Fritz-French C, Tyor W 2012 Interferon-α (IFNα) neurotoxicity. Cytokine Growth Factor Rev 23:7–14. doi:10.1016/j.cytogfr.2012.01.001. 22342642

[32] Sulkowski MS, Cooper C, Hunyady B, Jia J, Ogurtsov P, Peck-Radosavljevic M, Shiffman ML, Yurdaydin C, Dalgard O 2011 Management of adverse effects of Peg-IFN and ribavirin therapy for hepatitis C. Nat Rev Gastroenterol Hepatol 8:212–223. doi:10.1038/nrgastro.2011.21. 21386812

[33] Es-Saad S, Tremblay N, Baril M, Lamarre D 2012 Regulators of innate immunity as novel targets for panviral therapeutics. Curr Opin Virol 2:622–628. doi:10.1016/j.coviro.2012.08.009. 23017246 PMC7102864

[34] Du Y, Du T, Shi Y, Zhang A, Zhang C, Diao Y, Jin G, Zhou EM 2016 Synthetic toll-like receptor 7 ligand inhibits porcine reproductive and respiratory syndrome virus infection in primary porcine alveolar macrophages. Antiviral Res 131:9–18. doi:10.1016/j.antiviral.2016.04.005. 27079946

[35] Pattabhi S, Wilkins CR, Dong R, Knoll ML, Posakony J, Kaiser S, Mire CE, Wang ML, Ireton RC, Geisbert TW, Bedard KM, Iadonato SP, Loo YM, Gale M Jr 2015 Targeting innate immunity for antiviral therapy through small molecule agonists of the RLR pathway. J Virol 90:2372–2387. doi:10.1128/JVI.02202-15. 26676770 PMC4810700

[36] Chiang C, Beljanski V, Yin K, Olagnier D, Ben Yebdri F, Steel C, Goulet ML, Defilippis VR, Streblow DN, Haddad EK, Trautmann L, Ross T, Lin R, Hiscott J 2015 Sequence-specific modifications enhance the broad-spectrum antiviral response activated by RIG-I agonists. J Virol 89:8011–8025. doi:10.1128/JVI.00845-15. 26018150 PMC4505665

[37] Olagnier D, Scholte FEM, Chiang C, Albulescu IC, Nichols C, He Z, Lin R, Snijder EJ, van Hemert MJ, Hiscott J 2014 Inhibition of dengue and Chikungunya virus infections by RIG-I-mediated type I interferon-independent stimulation of the innate antiviral response. J Virol 88:4180–4194. doi:10.1128/JVI.03114-13. 24478443 PMC3993760

[38] Goulet ML, Olagnier D, Xu Z, Paz S, Belgnaoui SM, Lafferty EI, Janelle V, Arguello M, Paquet M, Ghneim K, Richards S, Smith A, Wilkinson P, Cameron M, Kalinke U, Qureshi S, Lamarre A, Haddad EK, Sekaly RP, Peri S, Balachandran S, Lin R, Hiscott J 2013 Systems analysis of a RIG-I agonist inducing broad spectrum inhibition of virus infectivity. PLoS Pathog 9:e1003298. doi:10.1371/journal.ppat.1003298. 23633948 PMC3635991

[39] Bedard KM, Wang ML, Proll SC, Loo YM, Katze MG, Gale M, Iadonato SP 2012 Isoflavone agonists of IRF-3 dependent signaling have antiviral activity against RNA viruses. J Virol 86:7334–7344. doi:10.1128/JVI.06867-11. 22532686 PMC3416323

[40] Guo F, Han Y, Zhao X, Wang J, Liu F, Xu C, Wei L, Jiang JD, Block TM, Guo JT, Chang J 2015 STING agonists induce an innate antiviral immune response against hepatitis B virus. Antimicrob Agents Chemother 59:1273–1281. doi:10.1128/AAC.04321-14. 25512416 PMC4335851

[41] Sali TM, Pryke KM, Abraham J, Liu A, Archer I, Broeckel R, Staverosky JA, Smith JL, Al-Shammari A, Amsler L, Sheridan K, Nilsen A, Streblow DN, Defilippis VR 2015 Characterization of a novel human-specific STING agonist that elicits antiviral activity against emerging alphaviruses. PLoS Pathog 11:e1005324. doi:10.1371/journal.ppat.1005324. 26646986 PMC4672893

[42] Akira S 2011 Innate immunity and adjuvants. Philos Trans R Soc Lond B Biol Sci 366:2748–2755. doi:10.1098/rstb.2011.0106. 21893536 PMC3146784

[43] Ortiz AL, Fuchs SY 2017 Anti-metastatic functions of type 1 interferons: foundation for the adjuvant therapy of cancer. Cytokine 89:4–11. doi:10.1016/j.cyto.2016.01.010. 26822709 PMC4959969

[44] Bresnahan WA, Hultman GE, Shenk T 2000 Replication of wild-type and mutant human cytomegalovirus in life-extended human diploid fibroblasts. J Virol 74:10816–10818. doi:10.1128/JVI.74.22.10816-10818.2000. 11044129 PMC110959

[45] Defilippis VR, Sali T, Alvarado D, White L, Bresnahan W, Früh KJ 2010 Activation of the interferon response by human cytomegalovirus occurs via cytoplasmic double-stranded DNA but not glycoprotein B. J Virol 84:8913–8925. doi:10.1128/JVI.00169-10. 20573816 PMC2919031

[46] Staeheli P, Dreiding P, Haller O, Lindenmann J 1985 Polyclonal and monoclonal antibodies to the interferon-inducible protein Mx of influenza virus-resistant mice. J Biol Chem 260:1821–1825. 3881442

[47] Pichlmair A, Lassnig C, Eberle CA, Górna MW, Baumann CL, Burkard TR, Bürckstümmer T, Stefanovic A, Krieger S, Bennett KL, Rülicke T, Weber F, Colinge J, Müller M, Superti-Furga G 2011 IFIT1 is an antiviral protein that recognizes 5′-triphosphate RNA. Nat Immunol 12:624–630. doi:10.1038/ni.2048. 21642987

[48] Seo JY, Yaneva R, Cresswell P 2011 Viperin: a multifunctional, interferon-inducible protein that regulates virus replication. Cell Host Microbe 10:534–539. doi:10.1016/j.chom.2011.11.004. 22177558 PMC3246677

[49] Chebath J, Benech P, Revel M, Vigneron M 1987 Constitutive expression of (2′−5′) oligo A synthetase confers resistance to picornavirus infection. Nature 330:587–588. doi:10.1038/330587a0. 2825034

[50] Phipps RP, Stein SH, Roper RL 1991 A new view of prostaglandin E regulation of the immune response. Immunol Today 12:349–352. doi:10.1016/0167-5699(91)90064-Z. 1958288

[51] Katsounas A, Schlaak JF, Lempicki RA 2011 CCL5: a double-edged sword in host defense against the hepatitis C virus. Int Rev Immunol 30:366–378. doi:10.3109/08830185.2011.593105. 22053974

[52] Baggiolini M, Walz A, Kunkel SL 1989 Neutrophil-activating peptide-1/interleukin 8, a novel cytokine that activates neutrophils. J Clin Invest 84:1045–1049. doi:10.1172/JCI114265. 2677047 PMC329758

[53] Hoge J, Yan I, Jänner N, Schumacher V, Chalaris A, Steinmetz OM, Engel DR, Scheller J, Rose-John S, Mittrücker HW 2013 IL-6 controls the innate immune response against Listeria monocytogenes via classical IL-6 signaling. J Immunol 190:703–711. doi:10.4049/jimmunol.1201044. 23241882

[54] Moulin E, Selby K, Cherpillod P, Kaiser L, Boillat-Blanco N 2016 Simultaneous outbreaks of dengue, Chikungunya and Zika virus infections: diagnosis challenge in a returning traveller with nonspecific febrile illness. New Microbes New Infect 11:6–7. doi:10.1016/j.nmni.2016.02.003. 27006779 PMC4786754

[55] Hamel R, Dejarnac O, Wichit S, Ekchariyawat P, Neyret A, Luplertlop N, Perera-Lecoin M, Surasombatpattana P, Talignani L, Thomas F, Cao-Lormeau VM, Choumet V, Briant L, Desprès P, Amara A, Yssel H, Missé D 2015 Biology of Zika virus infection in human skin cells. J Virol 89:8880–8896. doi:10.1128/JVI.00354-15. 26085147 PMC4524089

[56] White LK, Sali T, Alvarado D, Gatti E, Pierre P, Streblow D, Defilippis VR 2011 Chikungunya virus induces IPS-1-dependent innate immune activation and protein kinase R-independent translational shutoff. J Virol 85:606–620. doi:10.1128/JVI.00767-10. 20962078 PMC3014158

[57] Fros JJ, Pijlman GP 2016 Alphavirus infection: host cell shut-off and inhibition of antiviral responses. Viruses 8:166. doi:10.3390/v8060166. PMC4926186 27294951

[58] Ye J, Zhu B, Fu ZF, Chen H, Cao S 2013 Immune evasion strategies of flaviviruses. Vaccine 31:461–471. doi:10.1016/j.vaccine.2012.11.015. 23153447

[59] Akhrymuk I, Kulemzin SV, Frolova EI 2012 Evasion of the innate immune response: the Old World alphavirus nsP2 protein induces rapid degradation of Rpb1, a catalytic subunit of RNA polymerase II. J Virol 86:7180–7191. doi:10.1128/JVI.00541-12. 22514352 PMC3416352

[60] Yamamoto M, Sato S, Hemmi H, Hoshino K, Kaisho T, Sanjo H, Takeuchi O, Sugiyama M, Okabe M, Takeda K, Akira S 2003 Role of adaptor TRIF in the MyD88-independent toll-like receptor signaling pathway. Science 301:640–643. doi:10.1126/science.1087262. 12855817

[61] Defilippis VR, Alvarado D, Sali T, Rothenburg S, Früh K 2010 Human cytomegalovirus induces the interferon response via the DNA sensor ZBP1. J Virol 84:585–598. doi:10.1128/JVI.01748-09. 19846511 PMC2798427

[62] Sharma S, tenOever BR, Grandvaux N, Zhou GP, Lin R, Hiscott J 2003 Triggering the interferon antiviral response through an IKK-related pathway. Science 300:1148–1151. doi:10.1126/science.1081315. 12702806

[63] Feldman RI, Wu JM, Polokoff MA, Kochanny MJ, Dinter H, Zhu D, Biroc SL, Alicke B, Bryant J, Yuan S, Buckman BO, Lentz D, Ferrer M, Whitlow M, Adler M, Finster S, Chang Z, Arnaiz DO 2005 Novel small molecule inhibitors of 3-phosphoinositide-dependent kinase-1. J Biol Chem 280:19867–19874. doi:10.1074/jbc.M501367200. 15772071

[64] Diner EJ, Burdette DL, Wilson SC, Monroe KM, Kellenberger CA, Hyodo M, Hayakawa Y, Hammond MC, Vance RE 2013 The innate immune DNA sensor cGAS produces a noncanonical cyclic dinucleotide that activates human STING. Cell Rep 3:1355–1361. doi:10.1016/j.celrep.2013.05.009. 23707065 PMC3706192

[65] Grandvaux N, Servant MJ, Tenoever B, Sen GC, Balachandran S, Barber GN, Lin R, Hiscott J 2002 Transcriptional profiling of interferon regulatory factor 3 target genes: direct involvement in the regulation of interferon-stimulated genes. J Virol 76:5532–5539. doi:10.1128/JVI.76.11.5532-5539.2002. 11991981 PMC137057

[66] Noyce RS, Collins SE, Mossman KL 2006 Identification of a novel pathway essential for the immediate-early, interferon-independent antiviral response to enveloped virions. J Virol 80:226–235. doi:10.1128/JVI.80.1.226-235.2006. 16352547 PMC1317555

[67] Mata-Haro V, Cekic C, Martin M, Chilton PM, Casella CR, Mitchell TC 2007 The vaccine adjuvant monophosphoryl lipid A as a TRIF-biased agonist of TLR4. Science 316:1628–1632. doi:10.1126/science.1138963. 17569868

[68] Hiscott J 2007 Convergence of the NF-κB and IRF pathways in the regulation of the innate antiviral response. Cytokine Growth Factor Rev 18:483–490. doi:10.1016/j.cytogfr.2007.06.002. 17706453

[69] Pfeffer LM 2011 The role of nuclear factor κB in the interferon response. J Interferon Cytokine Res 31:553–559. doi:10.1089/jir.2011.0028. 21631354 PMC3128784

[70] Hoffmann HH, Schneider WM, Rice CM 2015 Interferons and viruses: an evolutionary arms race of molecular interactions. Trends Immunol 36:124–138. doi:10.1016/j.it.2015.01.004. 25704559 PMC4384471

[71] Sadler AJ, Williams BRG 2008 Interferon-inducible antiviral effectors. Nat Rev Immunol 8:559–568. doi:10.1038/nri2314. 18575461 PMC2522268

[72] Kavanagh D, McGlasson S, Jury A, Williams J, Scolding N, Bellamy C, Günther C, Ritchie D, Gale DP, Kanwar YS, Challis R, Buist H, Overell J, Weller B, Flossmann O, Blunden M, Meyer EP, Krucker T, Evans SJW, Campbell IL, Jackson AP, Chandran S, Hunt DPJ 2016 Type I interferon causes thrombotic microangiopathy by a dose-dependent toxic effect on the microvasculature. Blood 128:2824–2833. doi:10.1182/blood-2016-05-715987. 27663672 PMC5159705

[73] Coffman RL, Sher A, Seder RA 2010 Vaccine adjuvants: putting innate immunity to work. Immunity 33:492–503. doi:10.1016/j.immuni.2010.10.002. 21029960 PMC3420356

[74] Guo F, Zhao X, Gill T, Zhou Y, Campagna M, Wang L, Liu F, Zhang P, DiPaolo L, Du Y, Xu X, Jiang D, Wei L, Cuconati A, Block TM, Guo J-T, Chang J 2014 An interferon-beta promoter reporter assay for high throughput identification of compounds against multiple RNA viruses. Antiviral Res 107:56–65. doi:10.1016/j.antiviral.2014.04.010. 24792753 PMC4143146

[75] He Z, Zhu X, Wen W, Yuan J, Hu Y, Chen J, An S, Dong X, Lin C, Yu J, Wu J, Yang Y, Cai J, Li J, Li M 2016 Dengue virus subverts host innate immunity by targeting adaptor protein MAVS. J Virol 90:7219–7230. doi:10.1128/JVI.00221-16. 27252539 PMC4984625

[76] Dalrymple NA, Cimica V, Mackow ER 2015 Dengue virus NS proteins inhibit RIG-I/MAVS signaling by blocking TBK1/IRF3 phosphorylation: dengue virus serotype 1 NS4A is a unique interferon-regulating virulence determinant. mBio 6:e00553-15. doi:10.1128/mBio.00553-15. 25968648 PMC4436066

[77] Aguirre S, Maestre AM, Pagni S, Patel JR, Savage T, Gutman D, Maringer K, Bernal-Rubio D, Shabman RS, Simon V, Rodriguez-Madoz JR, Mulder LCF, Barber GN, Fernandez-Sesma A 2012 DENV inhibits type I IFN production in infected cells by cleaving human STING. PLoS Pathog 8:e1002934. doi:10.1371/journal.ppat.1002934. 23055924 PMC3464218

[78] Grant A, Ponia SS, Tripathi S, Balasubramaniam V, Miorin L, Sourisseau M, Schwarz MC, Sánchez-Seco MP, Evans MJ, Best SM, García-Sastre A 2016 Zika virus targets human STAT2 to inhibit type I interferon signaling. Cell Host Microbe 19:882–890. doi:10.1016/j.chom.2016.05.009. 27212660 PMC4900918

[79] Chattopadhyay S, Sen GC 2014 dsRNA-activation of TLR3 and RLR signaling: Gene induction-dependent and independent effects. J Interferon Cytokine Res 34:427–436. doi:10.1089/jir.2014.0034. 24905199 PMC4046345

[80] Beljanski V, Chiang C, Kirchenbaum GA, Olagnier D, Bloom CE, Wong T, Haddad EK, Trautmann L, Ross TM, Hiscott J 2015 Enhanced influenza virus-like particle vaccination with a structurally optimized RIG-I agonist as adjuvant. J Virol 89:10612–10624. doi:10.1128/JVI.01526-15. 26269188 PMC4580177

[81] Hochheiser K, Klein M, Gottschalk C, Hoss F, Scheu S, Coch C, Hartmann G, Kurts C 2016 Cutting edge: the RIG-I ligand 3pRNA potently improves CTL cross-priming and facilitates antiviral vaccination. J Immunol 196:2439–2443. doi:10.4049/jimmunol.1501958. 26819202

[82] Gray PM, Forrest G, Wisniewski T, Porter G, Freed DC, DeMartino JA, Zaller DM, Guo Z, Leone J, Fu TM, Vora KA 2012 Evidence for cyclic diguanylate as a vaccine adjuvant with novel immunostimulatory activities. Cell Immunol 278:113–119. doi:10.1016/j.cellimm.2012.07.006. 23121983

[83] Wang X, Majumdar T, Kessler P, Ozhegov E, Zhang Y, Chattopadhyay S, Barik S, Sen GC 2016 STING requires the adaptor TRIF to trigger innate immune responses to microbial infection. Cell Host Microbe 20:329–341. doi:10.1016/j.chom.2016.08.002. 27631700 PMC5026396

[84] Helbig KJ, Carr JM, Calvert JK, Wati S, Clarke JN, Eyre NS, Narayana SK, Fiches GN, McCartney EM, Beard MR 2013 Viperin is induced following dengue virus type-2 (DENV-2) infection and has anti-viral actions requiring the C-terminal end of viperin. PLoS Negl Trop Dis 7:e2178. doi:10.1371/journal.pntd.0002178. 23638199 PMC3630087

[85] Szretter KJ, Brien JD, Thackray LB, Virgin HW, Cresswell P, Diamond MS 2011 The interferon-inducible gene viperin restricts West Nile virus pathogenesis. J Virol 85:11557–11566. doi:10.1128/JVI.05519-11. 21880757 PMC3209274

[86] Jiang D, Weidner JM, Qing M, Pan XB, Guo H, Xu C, Zhang X, Birk A, Chang J, Shi PY, Block TM, Guo JT 2010 Identification of five interferon-induced cellular proteins that inhibit West Nile virus and dengue virus infections. J Virol 84:8332–8341. doi:10.1128/JVI.02199-09. 20534863 PMC2916517

[87] Perry ST, Buck MD, Lada SM, Schindler C, Shresta S 2011 STAT2 mediates innate immunity to dengue virus in the absence of STAT1 via the type I interferon receptor. PLoS Pathog 7:e1001297. doi:10.1371/journal.ppat.1001297. 21379341 PMC3040673

[88] Pinto AJ, Morahan PS, Brinton M, Stewart D, Gavin E 1990 Comparative therapeutic efficacy of recombinant interferons-alpha, -beta, and -gamma against alphatogavirus, bunyavirus, flavivirus, and herpesvirus infections. J Interferon Res 10:293–298. doi:10.1089/jir.1990.10.293. 1696607

[89] Errett JS, Suthar MS, McMillan A, Diamond MS, Gale M 2013 The essential, nonredundant roles of RIG-I and MDA5 in detecting and controlling West Nile virus infection. J Virol 87:11416–11425. doi:10.1128/JVI.01488-13. 23966395 PMC3807316

[90] Daffis S, Suthar MS, Szretter KJ, Gale M, Diamond MS 2009 Induction of IFN-beta and the innate antiviral response in myeloid cells occurs through an IPS-1-dependent signal that does not require IRF-3 and IRF-7. PLoS Pathog 5:e1000607. doi:10.1371/journal.ppat.1000607. 19798431 PMC2747008

[91] Ullah MO, Sweet MJ, Mansell A, Kellie S, Kobe B 2016 TRIF-dependent TLR signaling, its functions in host defense and inflammation, and its potential as a therapeutic target. J Leukoc Biol 100:27–45. doi:10.1189/jlb.2RI1115-531R. 27162325

[92] Ve T, Gay NJ, Mansell A, Kobe B, Kellie S 2012 Adaptors in toll-like receptor signaling and their potential as therapeutic targets. Curr Drug Targets 13:1360–1374. doi:10.2174/138945012803530260. 22664090

[93] Zhu J, Mohan C 2010 Toll-like receptor signaling pathways—therapeutic opportunities. Mediators Inflamm 2010:781235. doi:10.1155/2010/781235. 20981241 PMC2963142

[94] Yarilina A, DiCarlo E, Ivashkiv LB 2007 Suppression of the effector phase of inflammatory arthritis by double-stranded RNA is mediated by type I IFNs. J Immunol 178:2204–2211. doi:10.4049/jimmunol.178.4.2204. 17277125

[95] Vijay-Kumar M, Wu H, Aitken J, Kolachala VL, Neish AS, Sitaraman SV, Gewirtz AT 2007 Activation of toll-like receptor 3 protects against DSS-induced acute colitis. Inflamm Bowel Dis 13:856–864. doi:10.1002/ibd.20142. 17393379

[96] Sotolongo J, España C, Echeverry A, Siefker D, Altman N, Zaias J, Santaolalla R, Ruiz J, Schesser K, Adkins B, Fukata M 2011 Host innate recognition of an intestinal bacterial pathogen induces TRIF-dependent protective immunity. J Exp Med 208:2705–2716. doi:10.1084/jem.20110547. 22124111 PMC3244044

[97] Bhartiya D, Sklarsh JW, Maheshwari RK 1992 Enhanced wound healing in animal models by interferon and an interferon inducer. J Cell Physiol 150:312–319. doi:10.1002/jcp.1041500214. 1734035

[98] Lin Q, Wang L, Lin Y, Liu X, Ren X, Wen S, Du X, Lu T, Su SY, Yang X, Huang W, Zhou S, Wen F, Su SB 2012 Toll-like receptor 3 ligand polyinosinic:polycytidylic acid promotes wound healing in human and murine skin. J Invest Dermatol 132:2085–2092. doi:10.1038/jid.2012.120. 22572822

[99] Schwarz TF 2009 Clinical update of the AS04-adjuvanted human papillomavirus-16/18 cervical cancer vaccine, Cervarix. Adv Ther 26:983–998. doi:10.1007/s12325-009-0079-5. 20024678

[100] Hirotani T, Yamamoto M, Kumagai Y, Uematsu S, Kawase I, Takeuchi O, Akira S 2005 Regulation of lipopolysaccharide-inducible genes by MyD88 and Toll/IL-1 domain containing adaptor inducing IFN-beta. Biochem Biophys Res Commun 328:383–392. doi:10.1016/j.bbrc.2004.12.184. 15694359

[101] Kolanowski STHM, Dieker MC, Lissenberg-Thunnissen SN, van Schijndel GMW, van Ham SM, ten Brinke A 2014 TLR4-mediated pro-inflammatory dendritic cell differentiation in humans requires the combined action of MyD88 and TRIF. Innate Immun 20:423–430. doi:10.1177/1753425913498626. 23941760

[102] Hernandez A, Bohannon JK, Luan L, Fensterheim BA, Guo Y, Patil NK, McAdams C, Wang J, Sherwood ER 2016 The role of MyD88- and TRIF-dependent signaling in monophosphoryl lipid A-induced expansion and recruitment of innate immunocytes. J Leukoc Biol 100:1311–1322. doi:10.1189/jlb.1A0216-072R. 27354411 PMC5109999

[103] Kwissa M, Nakaya HI, Oluoch H, Pulendran B 2012 Distinct TLR adjuvants differentially stimulate systemic and local innate immune responses in nonhuman Primates. Blood 119:2044–2055. doi:10.1182/blood-2011-10-388579. 22246032 PMC3311246

[104] Sanin DE, Prendergast CT, Mountford AP 2015 IL-10 production in macrophages is regulated by a TLR-driven CREB-mediated mechanism that is linked to genes involved in cell metabolism. J Immunol 195:1218–1232. doi:10.4049/jimmunol.1500146. 26116503 PMC4505959

[105] Planès R, Ben Haij N, Leghmari K, Serrero M, BenMohamed L, Bahraoui E 2016 HIV-1 tat protein activates both the MyD88 and TRIF pathways to induce tumor necrosis factor alpha and interleukin-10 in human monocytes. J Virol 90:5886–5898. doi:10.1128/JVI.00262-16. 27053552 PMC4907244

[106] Boonstra A, Rajsbaum R, Holman M, Marques R, Asselin-Paturel C, Pereira JP, Bates EEM, Akira S, Vieira P, Liu YJ, Trinchieri G, O’Garra A 2006 Macrophages and myeloid dendritic cells, but not plasmacytoid dendritic cells, produce IL-10 in response to MyD88- and TRIF-dependent TLR signals, and TLR-independent signals. J Immunol 177:7551–7558. doi:10.4049/jimmunol.177.11.7551. 17114424

[107] Bowen WS, Minns LA, Johnson DA, Mitchell TC, Hutton MM, Evans JT 2012 Selective TRIF-dependent signaling by a synthetic toll-like receptor 4 agonist. Sci Signal 5:ra13–ra13. doi:10.1126/scisignal.2001963. 22337809 PMC3684200

[108] Mori N, Prager D 1997 Activation of the interleukin-10 gene in the human T lymphoma line HuT 78: identification and characterization of NF-kappa B binding sites in the regulatory region of the interleukin-10 gene. Eur J Haematol 59:162–170. doi:10.1111/j.1600-0609.1997.tb00970.x. 9310124

[109] Cao S, Zhang X, Edwards JP, Mosser DM 2006 NF-κB1 (p50) homodimers differentially regulate pro- and anti-inflammatory cytokines in macrophages. J Biol Chem 281:26041–26050. doi:10.1074/jbc.M602222200. 16835236 PMC2642587

[110] Chakrabarti A, Sadler AJ, Kar N, Young HA, Silverman RH, Williams BRG 2008 Protein kinase R-dependent regulation of interleukin-10 in response to double-stranded RNA. J Biol Chem 283:25132–25139. doi:10.1074/jbc.M804770200. 18625702 PMC2533086

[111] Takaoka A, Yanai H, Kondo S, Duncan G, Negishi H, Mizutani T, Kano S-I, Honda K, Ohba Y, Mak TW, Taniguchi T 2005 Integral role of IRF-5 in the gene induction programme activated by toll-like receptors. Nature 434:243–249. doi:10.1038/nature03308. 15665823

[112] Liu Q, Zhu Y, Yong WK, Sze NSK, Tan NS, Ding JL 2015 Cutting edge: synchronization of IRF1, JunB, and C/EBP activities during TLR3-TLR7 cross-talk orchestrates timely cytokine synergy in the proinflammatory response. J Immunol 195:801–805. doi:10.4049/jimmunol.1402358. 26109639 PMC4505950

[113] Botto S, Streblow DN, DeFilippis V, White L, Kreklywich CN, Smith PP, Caposio P 2011 IL-6 in human cytomegalovirus secretome promotes angiogenesis and survival of endothelial cells through the stimulation of survivin. Blood 117:352–361. doi:10.1182/blood-2010-06-291245. 20930069 PMC3037756

[114] Smith JL, Stein DA, Shum D, Fischer MA, Radu C, Bhinder B, Djaballah H, Nelson JA, Früh K, Hirsch AJ 2014 Inhibition of dengue virus replication by a class of small-molecule compounds that antagonize dopamine receptor D4 and downstream mitogen-activated protein kinase signaling. J Virol 88:5533–5542. doi:10.1128/JVI.00365-14. 24599995 PMC4019099

[115] Lanciotti RS, Kosoy OL, Laven JJ, Velez JO, Lambert AJ, Johnson AJ, Stanfield SM, Duffy MR 2008 Genetic and serologic properties of Zika virus associated with an epidemic, Yap State, Micronesia, 2007. Emerg Infect Dis 14:1232–1239. doi:10.3201/eid1408.080287. 18680646 PMC2600394

[116] Smith JL, Grey FE, Uhrlaub JL, Nikolich-Zugich J, Hirsch AJ 2012 Induction of the cellular microRNA, Hs_154, by West Nile virus contributes to virus-mediated apoptosis through repression of antiapoptotic factors. J Virol 86:5278–5287. doi:10.1128/JVI.06883-11. 22345437 PMC3347395

[117] Sanjana NE, Shalem O, Zhang F 2014 Improved vectors and genome-wide libraries for CRISPR screening. Nat Methods 11:783–784. doi:10.1038/nmeth.3047. 25075903 PMC4486245

[118] Livak KJ, Schmittgen TD 2001 Analysis of relative gene expression data using real-time quantitative PCR and the 2(−ΔΔ*C*_*T*_) method. Methods 25:402–408. doi:10.1006/meth.2001.1262. 11846609

