# Supplementary figures and images for: A Novel Agonist of the TRIF Pathway Induces a Cellular State Refractory to Replication of Zika, Chikungunya, and Dengue Viruses
Source: mBio. 2017 May 2;8(3):e00452-17. doi: 10.1128/mBio.00452-17 (PMC5414005; doi:10.1128/mBio.00452-17)

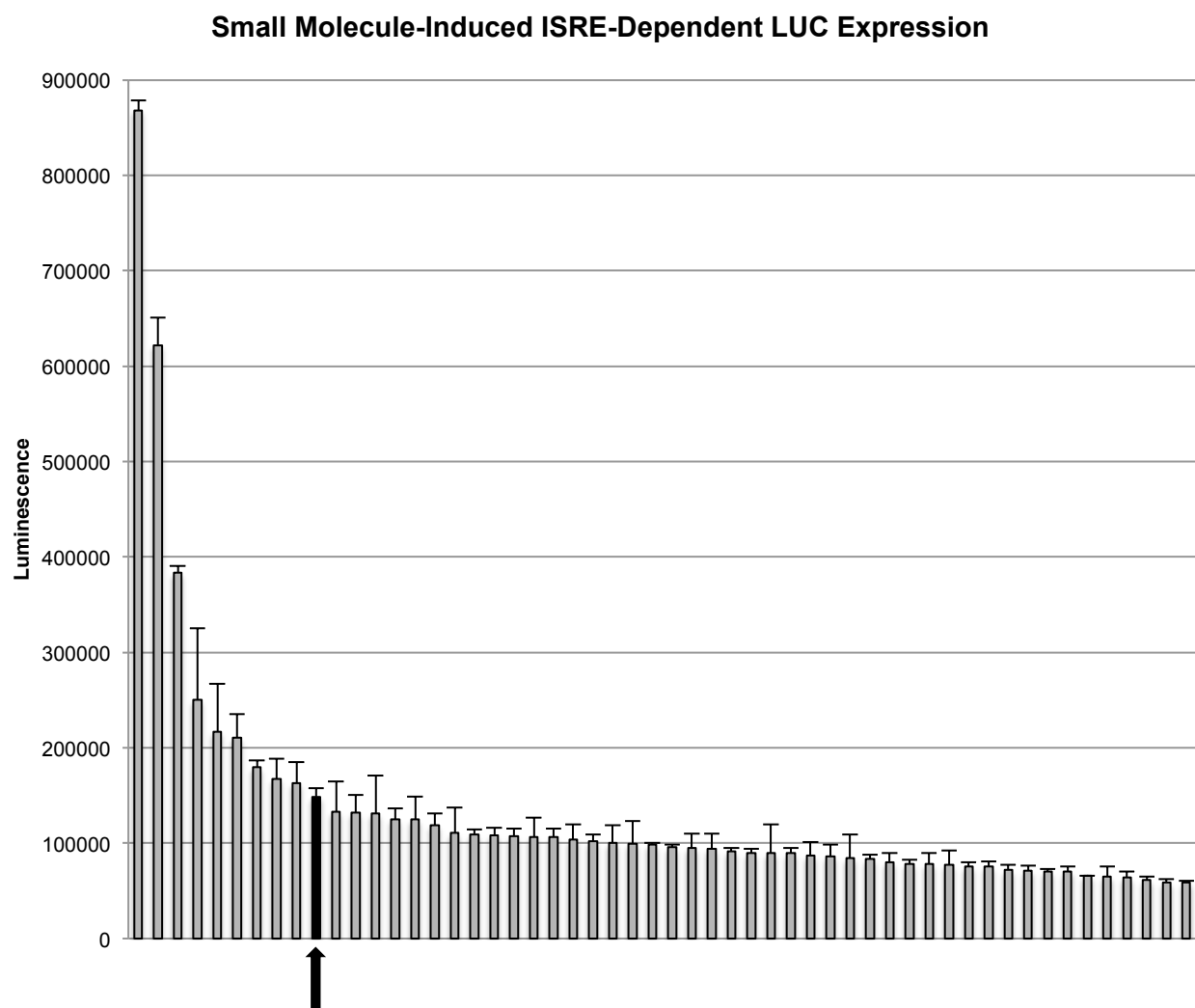

Supplement: FIG S1 [file mbo002173291sf1.pdf]
