# Supplementary material for: A Novel Agonist of the TRIF Pathway Induces a Cellular State Refractory to Replication of Zika, Chikungunya, and Dengue Viruses
Source: mBio. 2017 May 2;8(3):e00452-17. doi: 10.1128/mBio.00452-17 (PMC5414005; doi:10.1128/mBio.00452-17)
Supplement: FIG S2 [file mbo002173291sf2.pdf]

Supplemental Figure 2

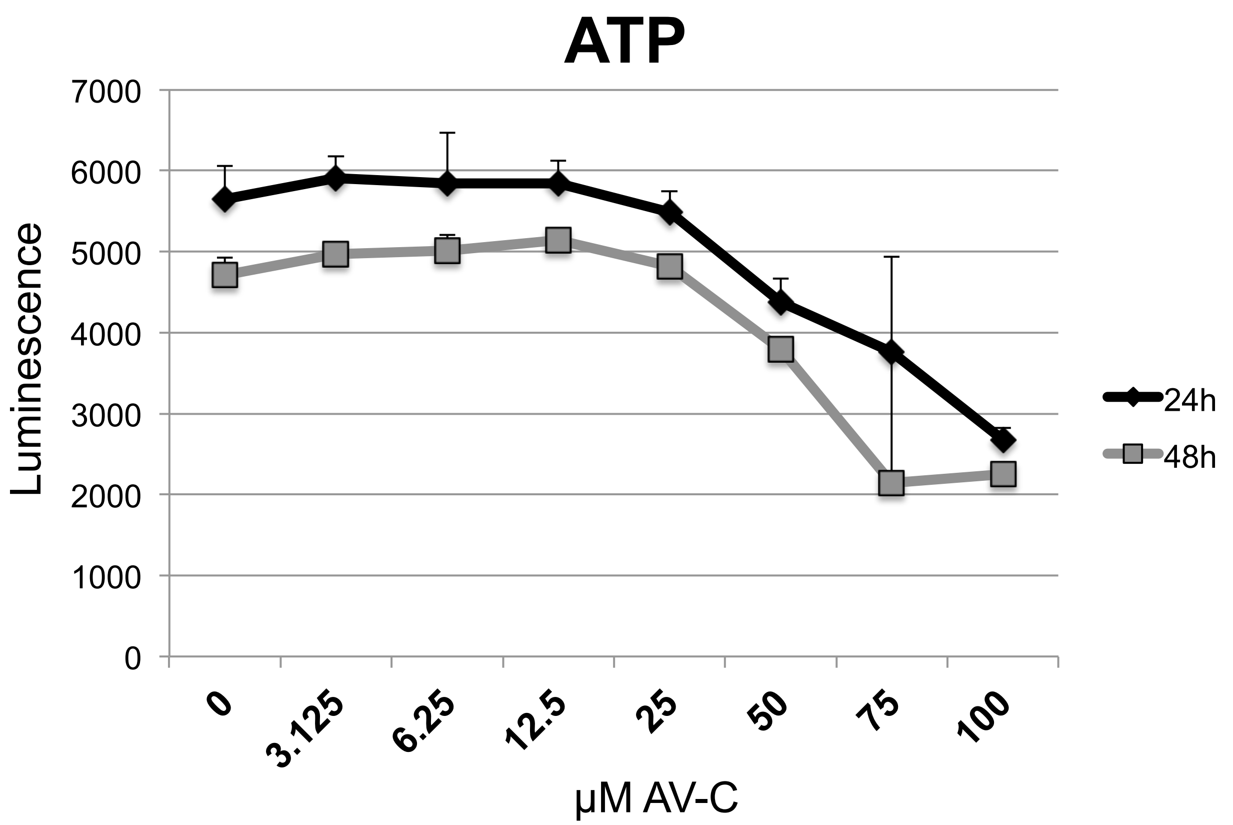

Supplemental Figure 2. ATP-dependent luminescence of THF following 24hr or 48hr exposure to indicated concentrations of AV-C (DMSO concentration normalized to 3%). Values displayed are raw luminescence values averaged from quadruplicate measurements ±SD following 24h or 48h exposure to indicated concentration of AV-C.
