# Supplementary material for: A Novel Agonist of the TRIF Pathway Induces a Cellular State Refractory to Replication of Zika, Chikungunya, and Dengue Viruses
Source: mBio. 2017 May 2;8(3):e00452-17. doi: 10.1128/mBio.00452-17 (PMC5414005; doi:10.1128/mBio.00452-17)
Supplement: FIG S3 [file mbo002173291sf3.pdf]

Supplemental Figure 3

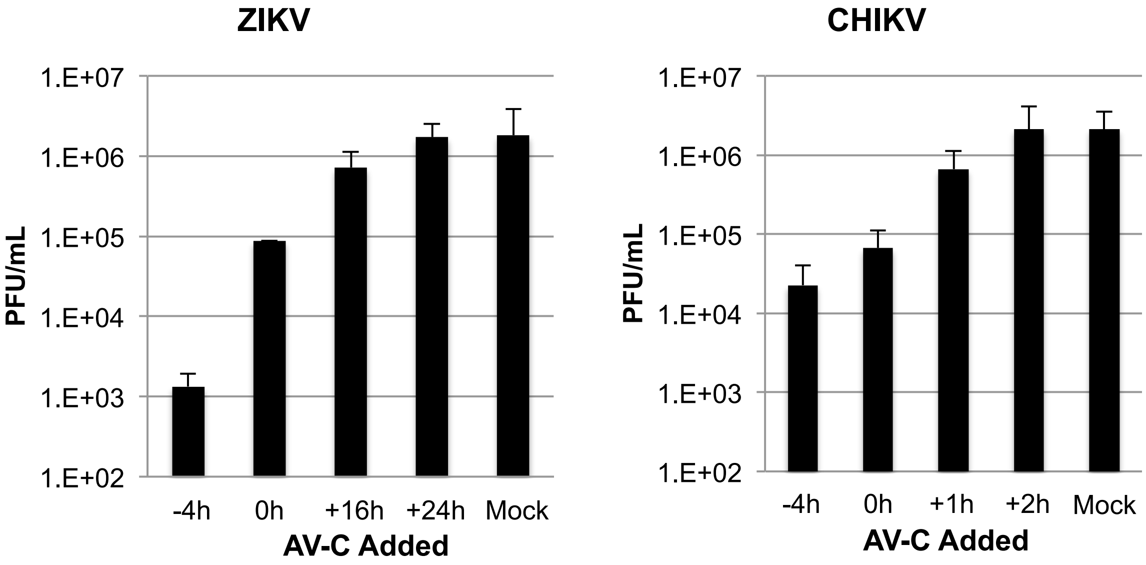

Supplemental Figure 3. Effect of AV-C Time of Addition on Virus Replication. Average PFU/mL ±SD of ZIKV and CHIKV grown on THF cells in triplicate in the presence of 12.5μM AV-C (DMSO concentration normalized to 1%) added to cells at indicated time pre- or post-infection. Media was harvested at 48h (CHIKV) or 72h (DENV, ZIKV) post infection and titered by serial dilution plaque (CHIKV) or focus forming unit (ZIKV, DENV) assay on Vero cells.
