# Supplementary material for: A Novel Agonist of the TRIF Pathway Induces a Cellular State Refractory to Replication of Zika, Chikungunya, and Dengue Viruses
Source: mBio. 2017 May 2;8(3):e00452-17. doi: 10.1128/mBio.00452-17 (PMC5414005; doi:10.1128/mBio.00452-17)
Supplement: FIG S4 [file mbo002173291sf4.pdf]

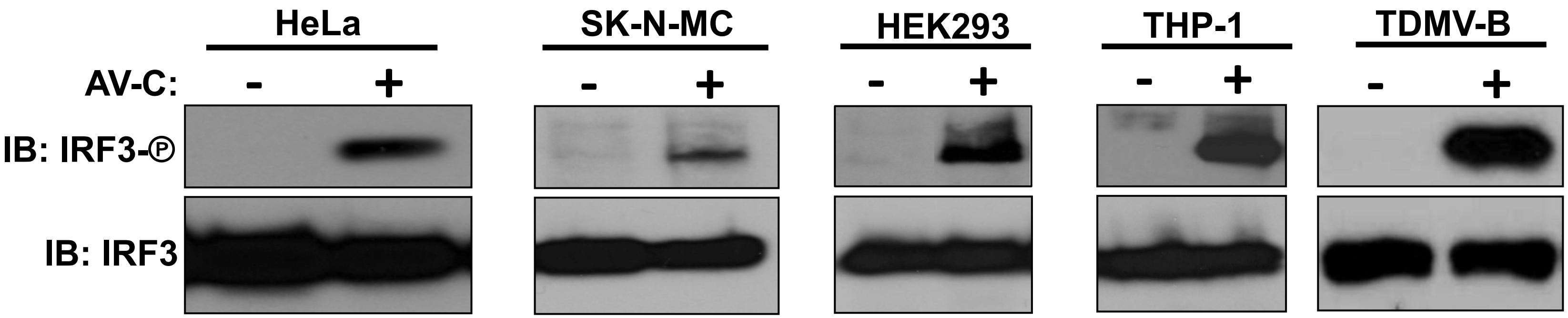

Supplemental Figure 4. Immunoblots showing phosphorylation status of IRF3 S386 and GAPDH loading control in HeLa, SK-N-MC, HEK293, THP-1, and TDMV-B cells either left untreated or following 4h treatment with 25μM AV-C.
