# Supplementary material for: A Novel Agonist of the TRIF Pathway Induces a Cellular State Refractory to Replication of Zika, Chikungunya, and Dengue Viruses
Source: mBio. 2017 May 2;8(3):e00452-17. doi: 10.1128/mBio.00452-17 (PMC5414005; doi:10.1128/mBio.00452-17)
Supplement: FIG S5 [file mbo002173291sf5.pdf]

Supplemental Figure 5

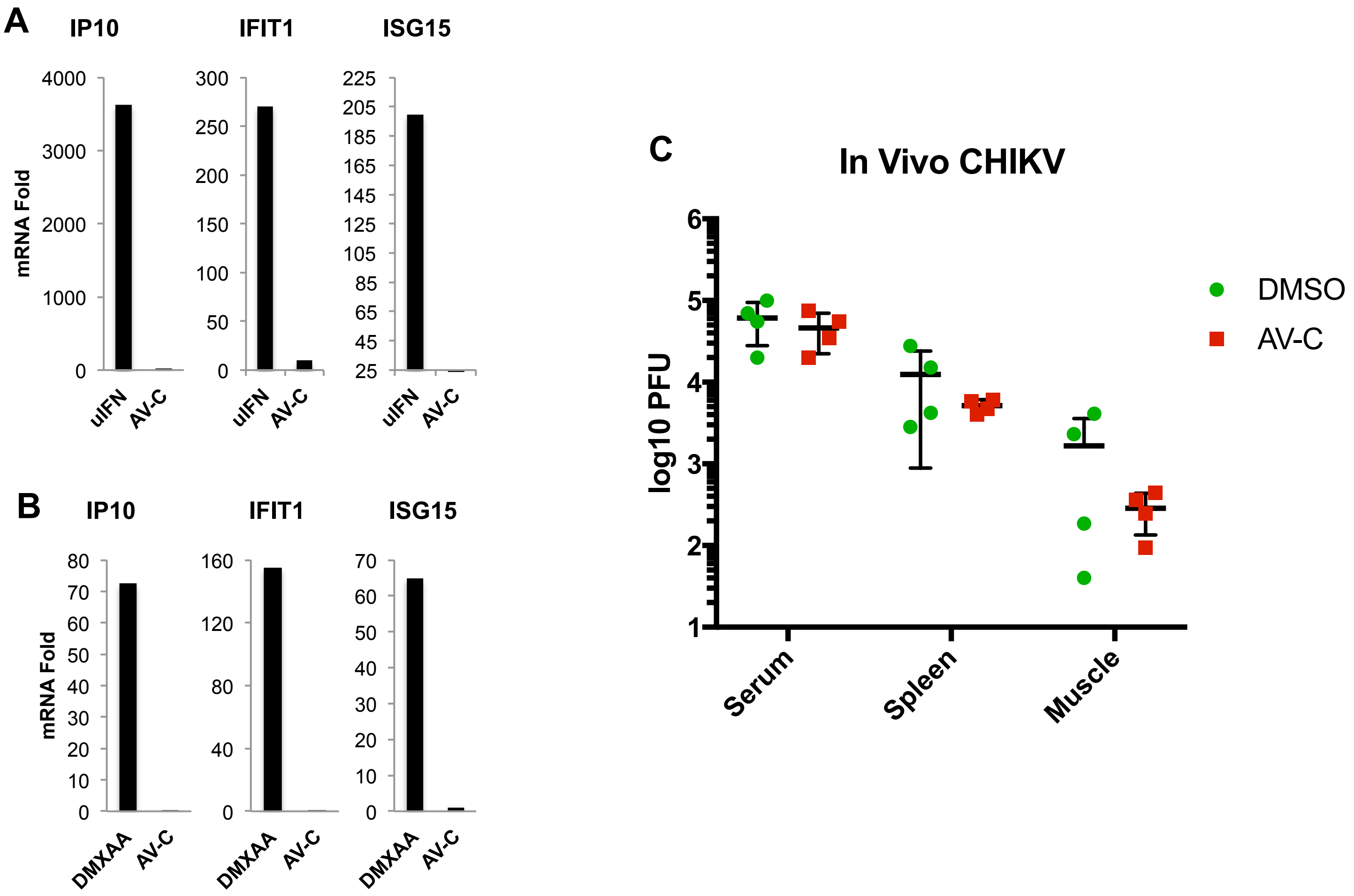

Supplemental Figure 5. In vivo activity of AV-C. **A.** Transcription of IFN-inducible genes IP10, IFIT1, and ISG15 in murine RAW264.7 macrophage-like cells treated for 8h with universal type I IFN (uIFN) or 25μM AV-C. Values displayed are average fold changes versus DMSO-treated cells of two biological replicates; **B.** Transcription of IP10, IFIT1, and ISG15 in murine RAW264.7 macrophage-like cells treated for 8h with serum harvested at 6h post treatment from C57BL/6J mice injected intraperitoneally with DMXAA or AV-C (25mg/kg). Values displayed are average fold changes versus DMSO-treated cells of two biological replicates; **C.** Average (±SD) CHIKV titers from homogenates of indicated tissues at 3d post infection from C57BL/6J mice (n = 4) treated intraperitoneally with DMSO or AV-C at 25mg/kg.
