# Supplementary material for: A Novel Agonist of the TRIF Pathway Induces a Cellular State Refractory to Replication of Zika, Chikungunya, and Dengue Viruses
Source: mBio. 2017 May 2;8(3):e00452-17. doi: 10.1128/mBio.00452-17 (PMC5414005; doi:10.1128/mBio.00452-17)
Supplement: FIG S6 [file mbo002173291sf6.pdf]

Supplemental Figure 6

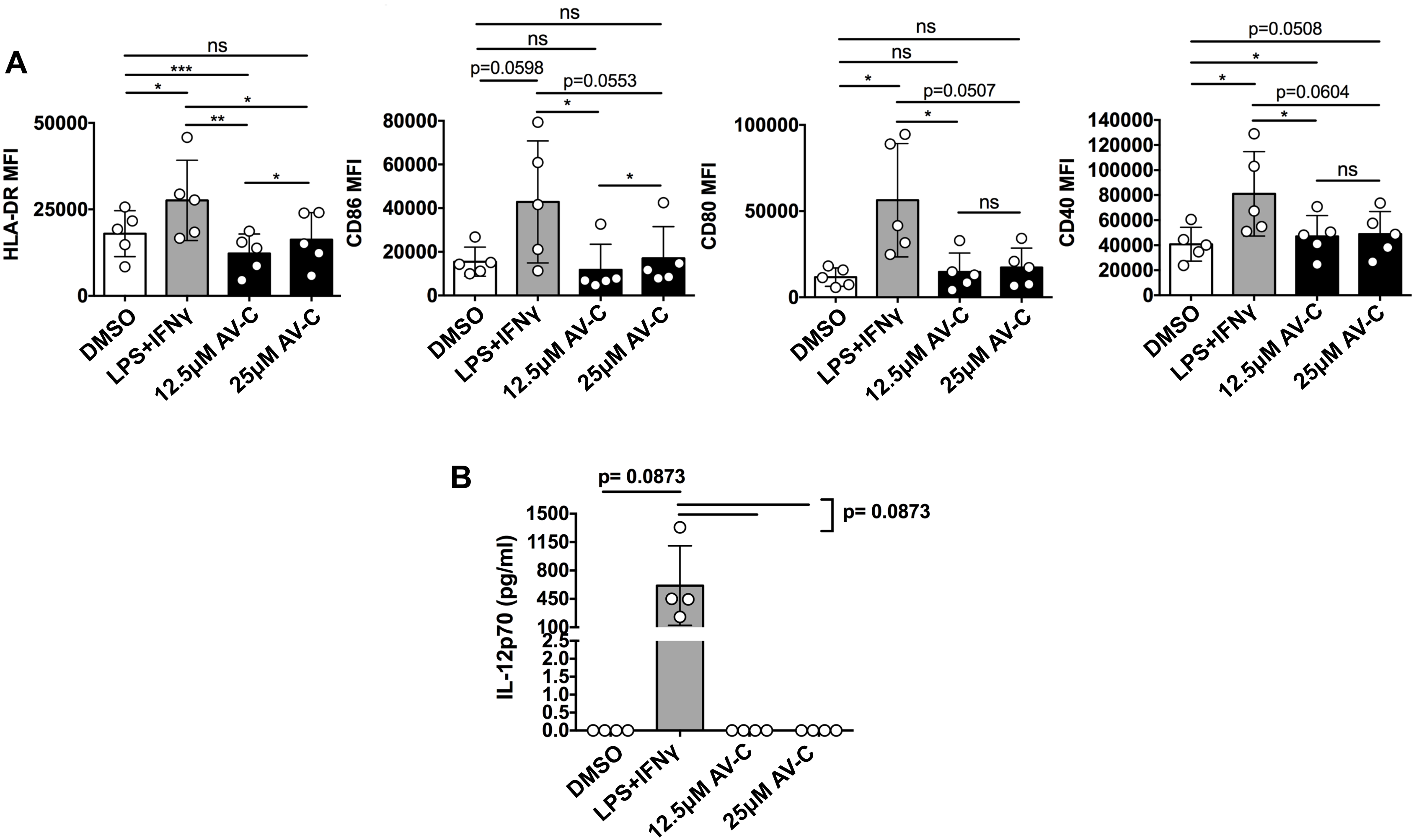

Supplemental Figure 6. AV-C does not induce maturation of myeloid dendritic cells or secretion of IL-12p70. A. Myeloid dendritic cells differentiated from healthy human PBMCs were treated with 1% DMSO or stimulated with 0.5µg/ml LPS + 40ng/ml IFNγ, 12.5uM or 25uM AV-C for 20h. DCs were harvested and analyzed by flow cytometry for the up-regulation of surface HLA-DR as well as the costimulatory molecules CD86, CD80 and CD40. Values presented are the average mean fluorescence intensity (MFI) ±SD of indicated marker from five individual donors (donor-specific values represented by clear circles); **B.** Myeloid dendritic cells differentiated from healthy human PBMCs were treated as in (**A**) for 20h. Culture supernatants were analyzed for the level of IL-12p70 by ELISA. Values presented are the average pg/ml ±SD from cells of four individual donors (donor-specific values represented by clear circles). Paired sample student's T-test comparisons were made between DMSO-treated and stimulated cells with ns= non significant, * = $p < 0.05$, ** = $p < 0.01$, *** $p < 0.001$.
