# Supplementary material for: A Novel Agonist of the TRIF Pathway Induces a Cellular State Refractory to Replication of Zika, Chikungunya, and Dengue Viruses
Source: mBio. 2017 May 2;8(3):e00452-17. doi: 10.1128/mBio.00452-17 (PMC5414005; doi:10.1128/mBio.00452-17)
Supplement: FIG S7 [file mbo002173291sf7.pdf]

Supplemental Figure 7

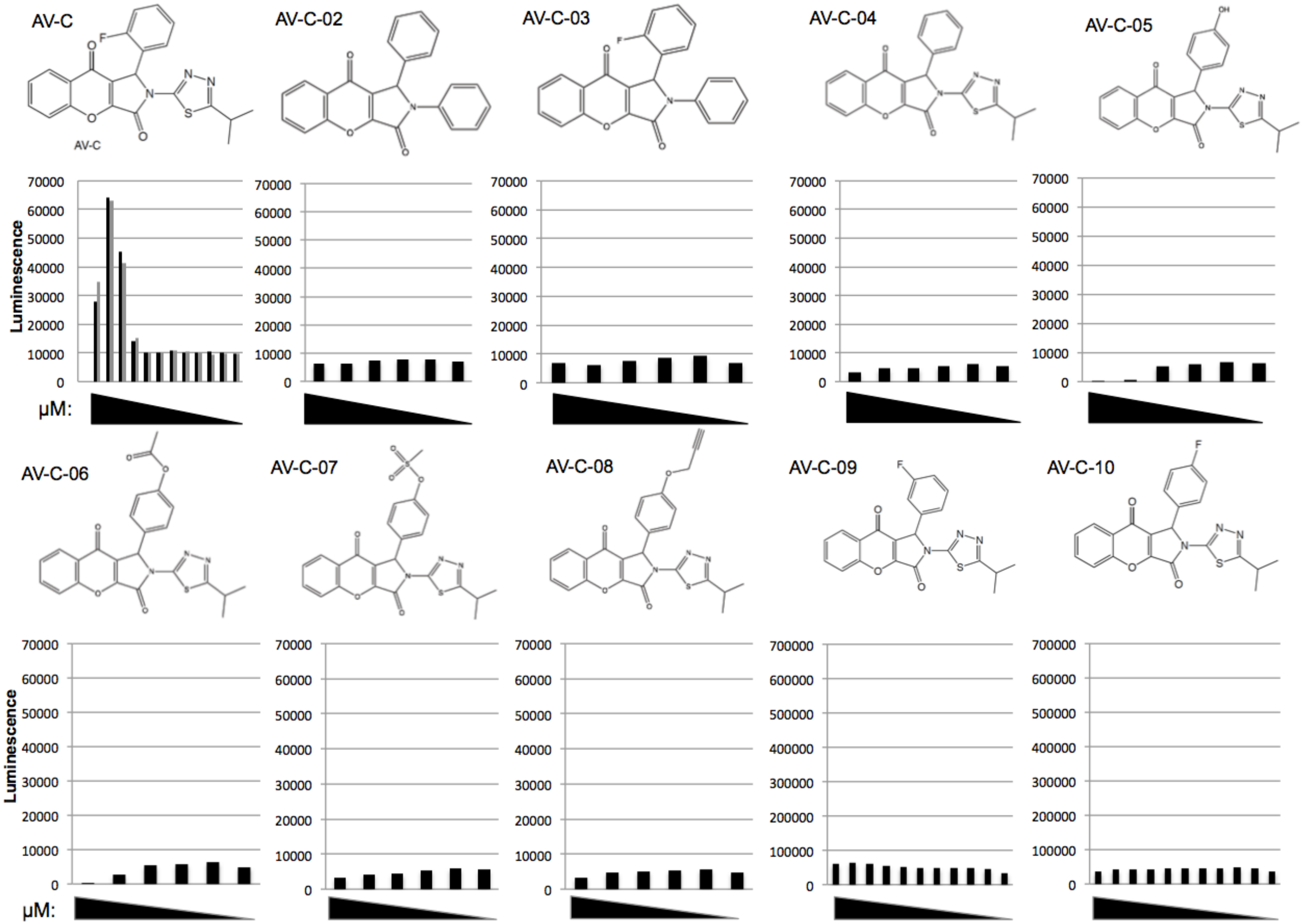

Supplemental Figure 7. Innate induction by AV-C analogs. Luminescence from THF-ISRE cells following 8h exposure to multiple concentrations of indicated AV-C derivative molecules.
