# Supplementary material for: A Novel Agonist of the TRIF Pathway Induces a Cellular State Refractory to Replication of Zika, Chikungunya, and Dengue Viruses
Source: mBio. 2017 May 2;8(3):e00452-17. doi: 10.1128/mBio.00452-17 (PMC5414005; doi:10.1128/mBio.00452-17)
Supplement: FIG S8 [file mbo002173291sf8.pdf]

Supplemental Figure 8

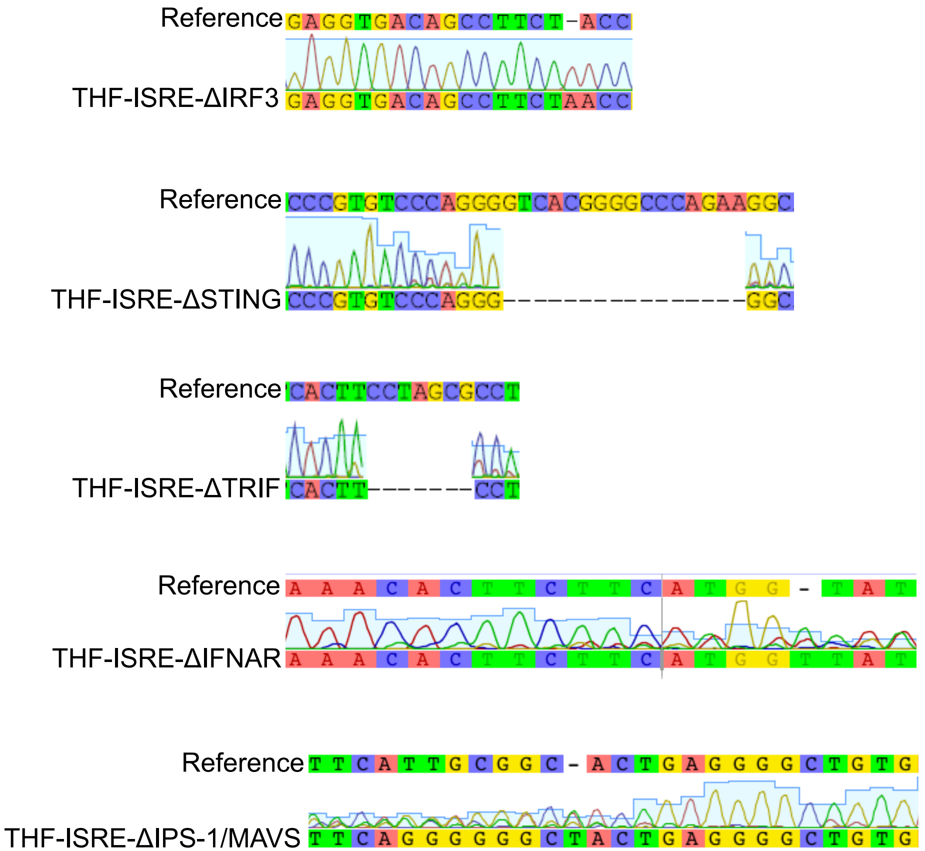

IRF3 gRNA: GAGGTGACAGCCTTCTACCG

STING gRNA: CCCGTGTCCCAGGGGTCACG

TRIF gRNA: CCTAGCGCCTTCGACATTCT

IFNAR gRNA: AAACACTTCTTCATGGTATG

IPS1 gRNA: AGTACTTCATTGCGGCACTG

Supplemental Figure 8. CRISPR/Cas9-mediated disruption of coding regions. A. Sanger sequencing electropherograms of genomic regions near gRNA targeting sites of indicated protein coding region.
